# Genetic Determinants of Lipids and Cardiovascular Disease Outcomes: A Wide-Angled Mendelian Randomization Investigation

**DOI:** 10.1161/CIRCGEN.119.002711

**Published:** 2019-12-17

**Authors:** Elias Allara, Gabriele Morani, Paul Carter, Apostolos Gkatzionis, Verena Zuber, Christopher N. Foley, Jessica M.B. Rees, Amy M. Mason, Steven Bell, Dipender Gill, Sara Lindström, Adam S. Butterworth, Emanuele Di Angelantonio, James Peters, Stephen Burgess, INVENT consortium

**Affiliations:** 1 Department of Public Health and Primary Care, BHF Cardiovascular Epidemiology Unit (E.A., P.C., J.M.B.R., A.M.M., S. Bell, A.S.B., E.D.A., J.P., S. Burgess), University of Cambridge, United Kingdom.; 2 National Institute for Health Research Blood and Transplant Research Unit in Donor Health and Genomics (E.A., S. Bell, A.S.B., E.D.A.), University of Cambridge, United Kingdom.; 3 MRC Biostatistics Unit (A.G., V.Z., C.N.F., S. Burgess), University of Cambridge, United Kingdom.; 4 National Institute for Health Research Cambridge Biomedical Research Centre (A.M.M, A.S.B., E.D.A), University of Cambridge, United Kingdom.; 5 Dipartimento di Scienze del Sistema Nervoso e del Comportamento, Università degli studi di Pavia, Italy (G.M.).; 6 Health Data Research UK, Cambridge, UK (A.S.B., E.D.A., J.P.).; 7 Department of Epidemiology and Biostatistics, Imperial College London (V.Z., D.G.).; 8 Edinburgh Clinical Trials Unit, Usher Institute of Population Health Sciences and Informatics, University of Edinburgh, United Kingdom (J.M.B.R.).; 9 Department of Epidemiology, University of Washington, Seattle (S.L.).

**Keywords:** aortic valve stenosis, epidemiology, lipids, Mendelian randomization, venous thromboembolism

## Abstract

Evidence from randomized trials has shown that therapies that lower LDL (low-density lipoprotein)-cholesterol and triglycerides reduce coronary artery disease (CAD) risk. However, there is still uncertainty about their effects on other cardiovascular outcomes. We therefore performed a systematic investigation of causal relationships between circulating lipids and cardiovascular outcomes using a Mendelian randomization approach.

**Methods:**

In the primary analysis, we performed 2-sample multivariable Mendelian randomization using data from participants of European ancestry. We also conducted univariable analyses using inverse-variance weighted and robust methods, and gene-specific analyses using variants that can be considered as proxies for specific lipid-lowering medications. We obtained associations with lipid fractions from the Global Lipids Genetics Consortium, a meta-analysis of 188 577 participants, and genetic associations with cardiovascular outcomes from 367 703 participants in UK Biobank.

**Results:**

For LDL-cholesterol, in addition to the expected positive associations with CAD risk (odds ratio [OR] per 1 SD increase, 1.45 [95% CI, 1.35–1.57]) and other atheromatous outcomes (ischemic cerebrovascular disease and peripheral vascular disease), we found independent associations of genetically predicted LDL-cholesterol with abdominal aortic aneurysm (OR, 1.75 [95% CI, 1.40–2.17]) and aortic valve stenosis (OR, 1.46 [95% CI, 1.25–1.70]). Genetically predicted triglyceride levels were positively associated with CAD (OR, 1.25 [95% CI, 1.12–1.40]), aortic valve stenosis (OR, 1.29 [95% CI, 1.04–1.61]), and hypertension (OR, 1.17 [95% CI, 1.07–1.27]), but inversely associated with venous thromboembolism (OR, 0.79 [95% CI, 0.67–0.93]) and hemorrhagic stroke (OR, 0.78 [95% CI, 0.62–0.98]). We also found positive associations of genetically predicted LDL-cholesterol and triglycerides with heart failure that appeared to be mediated by CAD.

**Conclusions:**

Lowering LDL-cholesterol is likely to prevent abdominal aortic aneurysm and aortic stenosis, in addition to CAD and other atheromatous cardiovascular outcomes. Lowering triglycerides is likely to prevent CAD and aortic valve stenosis but may increase thromboembolic risk.

## Abstract 2

Supplemental Digital Content is available in the text.

Evidence from randomized trials has shown that therapies that lower low-density lipoprotein (LDL)-cholesterol, such as statins, are beneficial for preventing or treating several atheromatous diseases such as coronary artery disease (CAD),^1^ ischemic stroke,^2^ and peripheral vascular disease,^3,4^ as well as both postoperative atrial fibrillation^5^ and heart failure in the presence of underlying CAD.^6,7^ However, for other cardiovascular outcomes, such as thromboembolic disease,^8^ hemorrhagic stroke,^9,10^ and aortic aneurysms,^11,12^ the effects of LDL-cholesterol lowering are less clear. Recently, the REDUCE-IT trial has shown that therapies that predominantly lower triglycerides can reduce major cardiovascular events.^13^ However, the effects of triglyceride lowering on nonatheromatous cardiovascular outcomes are largely unknown.

Although a randomized trial is the gold standard of evidence and is required to conclusively establish the effectiveness of a treatment, naturally occurring genetic variants can be used to help predict the outcome of a randomized trial in an approach known as Mendelian randomization (MR).^14^ For example, this approach has been used successfully to attest the effects of statins and predict the effects of PCSK9 (proprotein convertase subtilisin/kexin type 9) inhibitors on CAD risk seen in randomized trials.^15,16^ MR investigations have also shown positive associations of genetically predicted LDL-cholesterol with abdominal aortic aneurysm,^17^ ischemic stroke,^18^ and aortic stenosis,^19^ and positive associations of triglycerides with CAD.^20^ While MR makes strong assumptions including the genetic variants used in the analysis only influence the outcome via the stated risk factors,^21^ when both approaches can be undertaken MR has generally given results that agree with randomized trials.^22^

However, MR approaches have not yet been used to investigate the relationship between circulating lipids and some major cardiovascular conditions such as venous thromboembolism and heart failure. Additionally, approaches to estimate the independent effects of LDL-cholesterol, triglycerides, and high-density lipoprotein (HDL)-cholesterol on cardiovascular disease while accounting for genetic pleiotropy have only been performed for a limited set of outcomes.^17,19,20^

Our objective in this paper is to assess which cardiovascular outcomes could be treated by lipid-lowering therapies. We performed a systematic MR analysis of atheromatous and nonatheromatous cardiovascular disease outcomes. We considered disease outcomes in a single dataset (UK Biobank) to ensure a consistent approach to the analysis across different outcomes. We carried out polygenic analyses for each of LDL-cholesterol, HDL-cholesterol, and triglycerides, based on all common genetic variants associated with at least one of these risk factors, as well as gene-specific analyses based on variants in or near gene regions that mimic specific pharmaceutical interventions.

## Methods

### Data Availability

Summary statistics for the genetic associations with lipid fractions were taken from the Global Lipids Genetics Consortium, and are available at http://csg.sph.umich.edu/willer/public/lipids2013.^23,24^ Summary statistics for the genetic associations with outcomes were estimated in UK Biobank, and for replication with venous thromboembolism in the INVENT (International Network Against Venous Thrombosis) consortium (restricting to European descent participants and excluding participants from UK Biobank). Because of the sensitive nature of some of the data collected for this study, requests to access the datasets from qualified researchers trained in human subject confidentiality protocols may be sent to UK Biobank at https://www.ukbiobank.ac.uk/register-apply,^25^ and to the INVENT consortium by contacting the senior authors and Co-Conveners of the Consortium, Drs David-Alexandre Tregouet and Nicholas L. Smith.^26^

UK Biobank and all studies in these consortia were approved by their respective institutional review committee and their participants gave informed consent.

The methods are available in the Data Supplement.

## Results

### Participant Characteristics

Baseline characteristics of the participants in the UK Biobank are provided in Table 1. Around 46% of participants were men, and the mean age was 57 years. Around 10% were smokers, 93% were alcohol drinkers, and 4% had a history of diabetes mellitus at baseline.

**Table 1.**
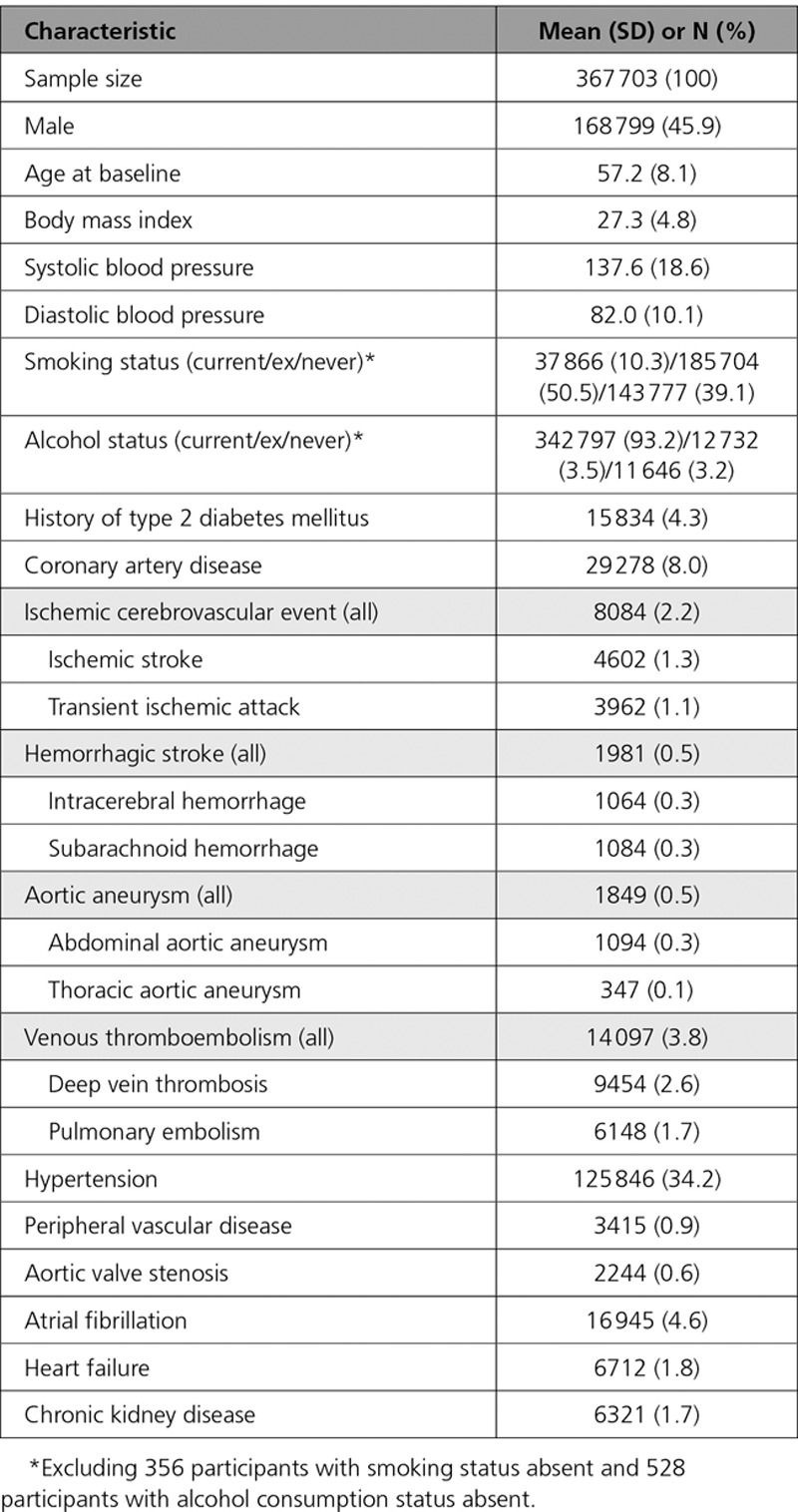
Baseline Characteristics of UK Biobank Participants Included in This Study and Numbers of Outcome Events

### Polygenic Analyses for All Lipid-Related Variants

**Figure 1.**
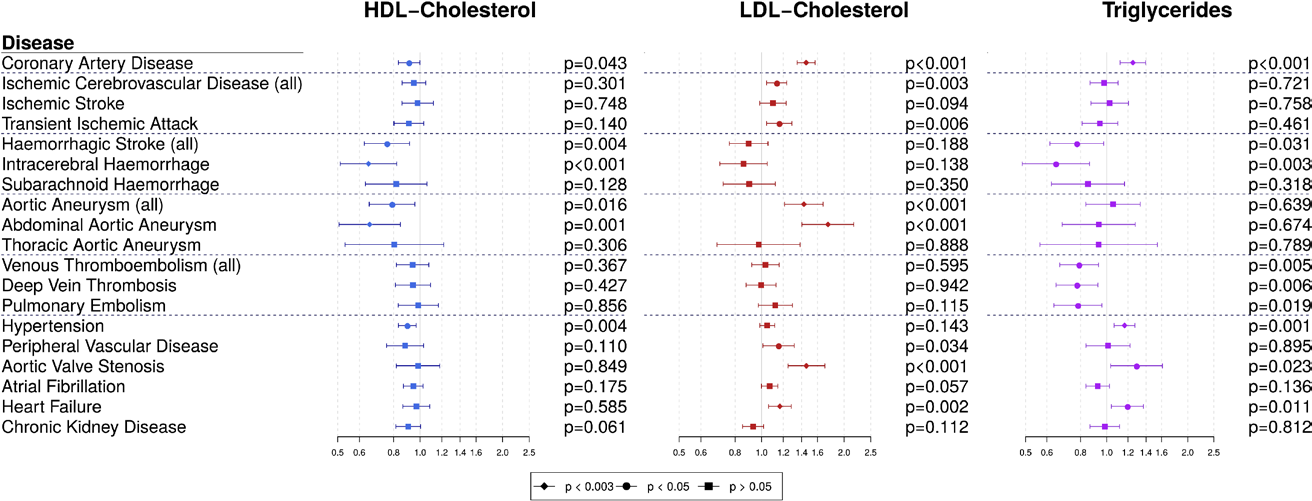
**Multivariable Mendelian randomization estimates (odds ratio with 95% confidence interval per 1 standard deviation increase in lipid fraction) from polygenic analyses including all lipid-associated variants.** HDL indicates high-density lipoprotein; and LDL, low-density lipoprotein.

Multivariable MR estimates are displayed graphically in Figure 1 and summarized in Table I in the Data Supplement. Estimates of heterogeneity between the causal estimates from different variants are provided in Tables II and III in the Data Supplement, and scatter plots of genetic associations for selected risk factor/outcome pairs are provided in Figure I in the Data Supplement. Significant heterogeneity was observed for several outcomes, although this is unsurprising given the number of genetic variants included in the analyses. Associations with the positive control outcome (CAD) were as expected for LDL-cholesterol and triglycerides. There were no associations with the negative control outcome (chronic kidney disease).

We found a strong association of genetically predicted LDL-cholesterol with CAD risk (odds ratio [OR] per 1 SD increase, 1.45 [95% CI, 1.35–1.57]), which was within the reference range of a previous study.^20^ The strongest associations by magnitude were for abdominal aortic aneurysm (OR, 1.75 [95% CI, 1.40–2.17), aortic valve stenosis (OR, 1.46 [95% CI, 1.25–1.70]), aortic aneurysm (OR, 1.43 [95% CI, 1.21–1.68]), and heart failure (OR, 1.17 [95% CI, 1.06–1.28]), which all met our threshold for statistical significance (*P*<0.003). We also observed a significant positive association for the combined outcome of ischemic stroke and transient ischemic attack (OR, 1.14 [95% CI, 1.04–1.24]). We saw positive associations at a nominal significance level (0.003 ≤ *P* < 0.05) for LDL-cholesterol with risk of transient ischemic attack and risk of peripheral vascular disease. The association with ischemic stroke was in the positive direction but nonsignificant (OR, 1.10 [95% CI, 0.98–1.23]).

Genetically predicted triglyceride levels were strongly associated with increased risk of coronary heart disease (CAD), consistent with previous results,^20^ as well as with increased risk of hypertension (OR, 1.17 [95% CI, 1.07–1.27]). Positive nominal associations were noted also for aortic valve stenosis and heart failure. A nominally significant inverse association was observed with hemorrhagic stroke, in particular with intracerebral hemorrhage (OR, 0.65 [95% CI, 0.49–0.86]). Inverse nominal associations were observed for triglycerides with all of the thromboembolic diseases we analyzed: deep vein thrombosis (OR, 0.78 [95% CI, 0.65–0.93]), pulmonary embolism (OR, 0.78 [95% CI, 0.64–0.96]), and any venous thromboembolism (OR, 0.79 [95% CI, 0.67–0.93]). A similar association with venous thromboembolism was estimated in a separate sample of 129 002 individuals of European ancestry from the INVENT consortium (OR, 0.84 [95% CI, 0.70–1.01]; *P*=0.057; Table 2). Genetically predicted HDL-cholesterol was nominally associated with lower risk of coronary heart disease (OR, 0.91 [95% CI, 0.83–1.00]) and hypertension and more strongly inversely associated with abdominal aortic aneurysm (OR, 0.65 [95% CI, 0.51–0.85]) and intracerebral hemorrhage (OR, 0.65 [95% CI, 0.51–0.82]). In exploratory analyses to investigate these findings, genetically predicted triglyceride levels were positively associated with tissue-type plasminogen activator but not with platelet count (Table IV in the Data Supplement). Positive associations were observed between genetically predicted triglycerides and both systolic and diastolic blood pressure, whereas inverse associations with HDL-cholesterol were not replicated (Table V in the Data Supplement).

**Table 2.**
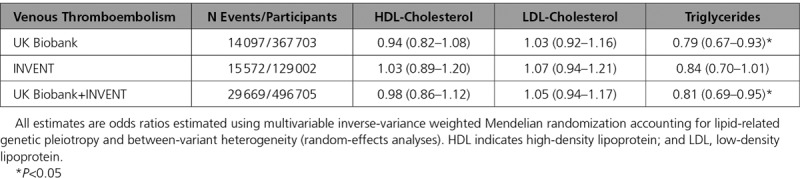
Replication and Meta-Analysis of the Association Between Genetically Predicted Triglycerides and Risk of Venous Thromboembolism in the INVENT Consortium (Odds Ratios with 95% Confidence Intervals)

Univariable MR estimates for each lipid risk factor in turn are displayed in Figures II through IV in the Data Supplement and summarized in Tables VI through VIII in the Data Supplement. Similar results were generally obtained from each of the univariable methods as for the multivariable methods, although CIs were slightly wider in some cases, especially for the MR-Egger method. The association between HDL-cholesterol and CAD became null in MR-Egger regression (OR, 0.99 [95% CI, 0.83–1.19]). Similarly, the associations of triglycerides with hemorrhagic stroke and its subtypes attenuated to the null in almost all univariable analyses (Table VIII in the Data Supplement). Another notable difference was a positive association of genetically predicted triglycerides with increased aortic aneurysm risk, particularly for abdominal aortic aneurysm. While the association with aortic aneurysm was not strongly apparent in the multivariable analysis, it was evident for each of the univariable analysis methods.

We also assessed whether the associations of genetically predicted lipids with outcomes that are comorbid with CAD (eg, heart failure) may be mediated via CAD, and similarly for outcomes that are comorbid with type 2 diabetes mellitus (T2D), for example, hypertension. On adjustment for CAD risk (Table 3), the associations of genetically predicted LDL-cholesterol and triglycerides with heart failure attenuated sharply, suggesting that their effects on heart failure might be mediated via CAD. As a negative control, we also performed the same analysis for abdominal aortic aneurysm. The association with genetically predicted LDL-cholesterol attenuated slightly but was still clearly positive. We also performed multivariable MR analyses excluding participants with CAD for heart failure (2383 remaining cases). This analysis gave null estimates for all lipid fractions (Table IX in the Data Supplement). This suggests that LDL-cholesterol is unlikely to be a causal risk factor for heart failure where there is no comorbidity with CAD. On adjustment for body mass index and T2D mellitus (Table X in the Data Supplement), associations between lipids and hypertension, abdominal aortic aneurysm, and hemorrhagic stroke did not change substantially compared with unadjusted analyses, suggesting that neither body mass index nor T2D mellitus is likely to mediate the effects of lipids on these outcomes. While adjusting for heart failure, heart failure was strongly associated with venous thromboembolism, but associations between lipids and venous thromboembolism did not change markedly (Table X in the Data Supplement). This confirms that heart failure is an independent risk factor for venous thromboembolism but suggests it is unlikely to mediate the association between triglycerides and venous thromboembolism.

**Table 3.**
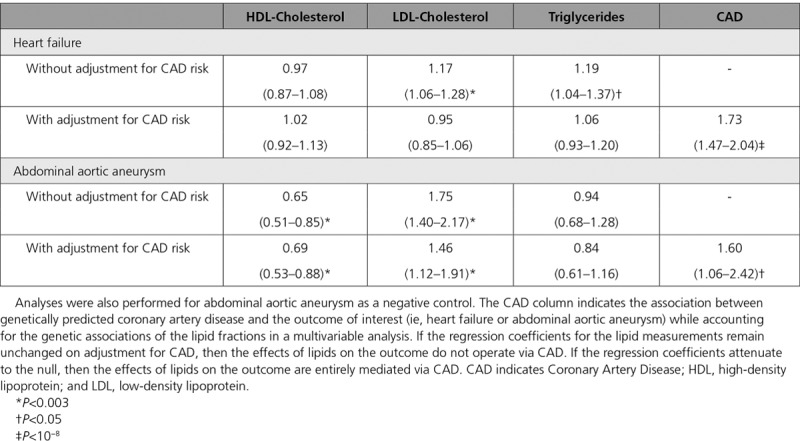
Multivariable Mendelian Randomization Estimates (Odds Ratio With 95% CI) for Heart Failure Without and With Adjustment for CAD Risk

### Gene-Specific Analyses for Drug Proxy Variants

**Figure 2.**
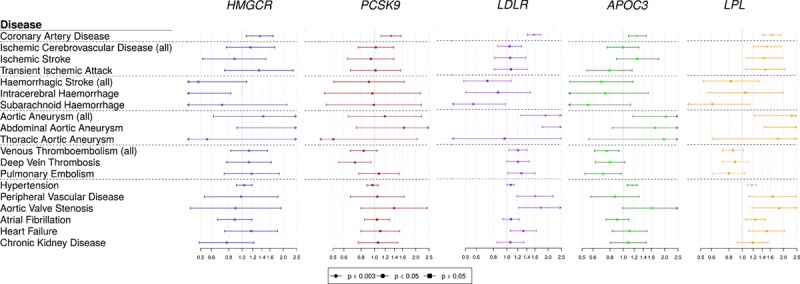
**Univariable Mendelian randomization estimates (odds ratio with 95% CI per 1 SD increase in lipid fraction) for variants in specific gene regions.** Estimates are scaled to a unit SD increase in LDL-cholesterol for the *HMGCR*, *PCSK9*, and *LDLR* regions, and to a SD increase in triglycerides for the *APOC3* and *LPL* regions.

MR estimates for specific gene regions are displayed graphically in Figure 2 and summarized in Tables XI and XII in the Data Supplement. All the gene regions analyzed showed clear associations with CAD risk, confirming their involvement in cardiovascular disease etiology and the relevance of existing and proposed lipid-lowering therapies for CAD risk reduction.

Results for the low-density lipoprotein receptor (*LDLR*) gene regions, and to a lesser extent for 3-hydroxy-3-methylglutaryl-CoA reductase (*HMGCR*) and *PCSK9*, were similar to those for LDL-cholesterol in the polygenic analyses, although with wider confidence intervals. Significant positive associations were obtained for *LDLR* with aortic and abdominal aortic aneurysm, venous thromboembolism, aortic valve stenosis, and heart failure, and an inverse association was observed for subarachnoid hemorrhage. Variants in the *HMGCR* gene region were inversely associated with intracerebral hemorrhage (*P*=0.022). Results for apolipoprotein C3 (*APOC3*) and lipoprotein lipase (*LPL*) followed a pattern more similar to the analysis for triglycerides, showing inverse associations with thromboembolic diseases (*P*=0.007 for *APOC3*, *P*=0.089 for *LPL* for any venous thromboembolism). In both regions, associations with aortic aneurysm, aortic valve stenosis and hypertension were all positive. Additionally, variants in the *LPL* region were also positively associated with all ischemic cerebrovascular diseases.

## Discussion

In this study, we assessed the causal role of 3 major lipid fractions for a range of cardiovascular diseases in a large population-based cohort using the principle of MR while accounting for genetic pleiotropy between the lipid measures. Our most notable findings were the associations between genetically predicted triglycerides and decreased risk of thromboembolic diseases both in polygenic analyses and in gene-specific analyses for the *APOC3* gene region, suggesting that reducing triglycerides may increase risk for venous thromboembolism. Additionally, as summarized in Table XIII in the Data Supplement, we found: (1) evidence supporting the current understanding of the cause of CAD, suggesting independent causal roles for LDL-cholesterol and triglycerides, both in the polygenic analyses and for all the drug-related gene-specific analyses^20,27^; (2) positive associations of genetically predicted LDL-cholesterol with abdominal aortic aneurysm and aortic valve stenosis, as well as atheromatous cardiovascular outcomes that are already addressed in clinical guidelines (eg, peripheral vascular disease and the combined outcome of ischemic stroke and transient ischemic attack); (3) positive associations of genetically predicted LDL-cholesterol and triglycerides with heart failure that appear to be mediated by CAD; (4) associations between genetically predicted triglycerides and increased risk of aortic stenosis and hypertension; and (5) inverse associations between genetically predicted HDL-cholesterol and abdominal aortic aneurysm and hemorrhagic stroke (in particular, intracerebral hemorrhage) that seem to be independent of body mass index/type 2 diabetes mellitus.

The importance of these findings is 3-fold. First, they help us to better understand the etiology and pathophysiology of common cardiovascular outcomes, and so to identify additional potential indications for lipid-lowering therapies. Second, association estimates provided in this paper can inform calculations on the risk-benefit and cost-benefit of these therapies. Third, these findings can aid identification of which patients would most benefit from (or should avoid) lipid-lowering therapies.

Genetic predisposition to lower triglycerides was associated with an increased risk of thromboembolic diseases. In contrast, previous observational evidence did not suggest an association between triglycerides and venous thrombosis,^28,29^ although other lipid measures (in particular apolipoprotein B and lipoprotein[a]) were inversely associated with venous thrombosis mortality in a meta-analysis of over 700 000 participants from the Emerging Risk Factors Collaboration.^29^ Our result was consistent across 3 of the 4 MR methods and was replicated using data from the INVENT consortium, an independent data source for genetic associations with the outcome. Associations with thromboembolic outcomes using the MR-Egger method were slightly attenuated and compatible with the null, raising the possibility of unmeasured genetic pleiotropy, although this method generally has lower power compared with the conventional inverse-variance weighted method.^30^ Variants in the *APOC3* gene region were associated with thromboembolic events, suggesting that lowering triglyceride levels via intervening on this pathway may increase thromboembolism risk. Additionally, the positive genetic association between triglycerides and tissue-type plasminogen activator reported here confirms the findings of a previous observational study among 1227 men free of coronary heart disease.^31^ However, genetically predicted triglycerides were not associated with platelet count. This is in contrast to the APPROACH trial, a trial of triglyceride-lowering therapy in patients with familial chylomicronemia, in which thrombocytopenia led to excess early terminations among patients with familial chylomicronemia^32^ but in line with (1) 2 other trials of APOC3 inhibitors^33,34^ and (2) with the fluctuations in platelet counts (both thrombocytopenia and thrombocytosis) noted in an observational study of 86 patients with familial chylomicronemia.^35^ Further investigation is needed to confirm the unexpected genetic association of triglycerides with venous thromboembolism.

Both the positive association between genetic predictors of LDL-cholesterol and abdominal aortic aneurysm, and the inverse association for HDL-cholesterol, replicate a previous MR study which did not include UK Biobank participants.^17^ This evidence is complemented by a randomized trial that has shown benefit from screening and treating abdominal aortic aneurysm using various interventions including statins.^11^ No significant association was seen with thoracic aortic aneurysm, in line with the different pathophysiology of the 2 disease subtypes.^36^ Overall, these findings provide further support to the hypothesis that increased LDL-cholesterol has deleterious effects on abdominal aortic aneurysm and suggest that LDL-cholesterol lowering therapies may be beneficial in preventing abdominal aortic aneurysm.

Similarly, the positive association between genetically predicted LDL-cholesterol and aortic valve stenosis replicates a previous MR study.^19^ We also demonstrated a positive association for triglycerides, in line with the previous study (although their result did not achieve conventional levels of statistical significance). Our association was robust to all sensitivity analyses and evidenced in gene-specific analyses for both the *APOC3* and *LPL* loci. These findings are consistent with previous observational and pathological studies suggesting a role of atherosclerotic processes in early valve lesions^37,38^ whereas clinical trials showed no benefit from LDL-cholesterol lowering via statins on aortic stenosis progression.^39^ Taken together, this suggests that increased LDL-cholesterol and triglycerides may facilitate initiation of early lesions, but that lipid-lowering therapies may be ineffective in preventing the progression of aortic stenosis.

CAD is a well-known risk factor for heart failure,^40^ as myocardial ischemic damage reduces myocardial contractility and ventricular function. Associations of heart failure with genetically predicted LDL-cholesterol and triglycerides disappeared after adjusting for CAD. The association was also absent when omitting participants with a CAD diagnosis from analyses. In contrast, associations with abdominal aortic aneurysm attenuated only slightly after adjustment for CAD. Overall, these results suggest that lipid-lowering therapies are only likely to influence risk of heart failure via their effects on CAD.

Our analyses also identified inverse associations of genetically predicted HDL-C and triglyceride levels with risk of intracerebral hemorrhage (albeit the latter association was not robust to most sensitivity analyses), consistent with a recent observational analysis for triglycerides.^41^ Furthermore, we identified a detrimental effect of HMGCR inhibition on intracerebral hemorrhage risk, in keeping with a large randomized trial of atorvastatin in patients with recent transient ischemic attack or stroke, which found statin therapy to lower risk of major cardiovascular events but increase the risk of hemorrhagic stroke.^42^ However, this finding has not been replicated in large meta-analyses.^43^ The mechanism relating lipid traits with risk of intracerebral hemorrhage requires further exploration.

Finally, the inverse association between HDL-cholesterol and CAD was only nominally significant in the main multivariable analyses but became null with MR-Egger regression, suggesting that HDL-cholesterol is unlikely to be a causal risk factor for CAD.

Our investigation has both strengths and limitations. The large sample size of over 360 000 participants and the broad set of outcomes analyzed render this one of the most comprehensive MR analyses on cardiovascular disease conducted to date. Availability of multiple cardiovascular conditions within the same study enabled cross-comparisons between diseases and enabled us to perform adjusted analyses in the same participants and to assess mediation of causal effects. This investigation has a number of limitations. Lack of publicly available summary data from external datasets prevented us from performing replication analyses for some associations, for example, those for heart failure. This design still allows sensitivity analyses such as MR-Egger and the weighted median estimator which enabled us to investigate and account for the possible presence of genetic pleiotropy and gene-specific analyses that are less likely to be influenced by pleiotropy as they only include variants from a single gene region where the function is well known. Significant heterogeneity was observed in the polygenic analysis for several outcomes, suggesting that some variants may have pleiotropic effects. Additionally, this investigation was conducted in UK-based middle- to late-aged participants of European ancestries. While this is recommended for MR to ensure that genetic associations are not influenced by population stratification, it means that results may not be generalizable to other ethnicities or nationalities.

In conclusion, multivariable MR analyses accounting for lipid-related genetic pleiotropy support the hypothesis that circulating lipids have causal effects on a wide range of cardiovascular diseases. Interventions that lower LDL-cholesterol are likely to be beneficial in preventing aortic aneurysm and aortic stenosis in addition to several other atheromatous diseases. Triglyceride-lowering treatments are likely to be beneficial in preventing CAD and aortic valve stenosis, but caution is needed due to the possibility of increased risk of venous thromboembolism.

## Acknowledgments

The research has been conducted using the UK Biobank Resource under Application Number 26865.

## Sources of Funding

The study’s coordinating center has been underpinned by grants G0800270, MR/L003120/1 and MC_UU_12013/3 from the UK Medical Research Council, grants SP/09/002, RG/08/014, and RG13/13/30194 from the British Heart Foundation, grants from the National Institute for Health Research (NIHR) through the Cambridge Biomedical Research Centre, and grant HEALTH-F2-2012-279233 from the European Commission Framework 7 through the EPIC-CVD award. The NIHR Blood and Transplant Research Unit (BTRU) in Donor Health and Genomics is supported by grant NIHR BTRU-2014-10024. Dr Burgess is supported by a Sir Henry Dale Fellowship jointly funded by the Wellcome Trust and the Royal Society (grant number 204623/Z/16/Z). Dr Allara is supported by a NIHR BTRU PhD Studentship. Dr Gill is funded by the Wellcome 4i Clinical PhD Programme at Imperial College London. Dr Peters was funded by a UKRI Innovation Fellowship (MR/S004068/1). This work has received support from the EU/EFPIA Innovative Medicines Initiative (https://www.imi.europa.eu/) Joint Undertaking BigData@Heart grant no. 116074. Aspects of the analysis were supported by the Cambridge Substantive Site of Health Data Research UK and the National Institute for Health Research (Cambridge Biomedical Research Centre at the Cambridge University Hospitals NHS Foundation Trust (The views expressed are those of the authors and not necessarily those of the NHS, the NIHR or the Department of Health and Social Care. The funding sources had no role in the design and conduct of the study; collection, management, analysis, and interpretation of the data; preparation, review, or approval of the manuscript; and decision to submit the manuscript for publication.

## Disclosures

None.

## Supplementary Material
