# Supplementary material for: Genetic Determinants of Lipids and Cardiovascular Disease Outcomes: A Wide-Angled Mendelian Randomization Investigation
Source: Circ Genom Precis Med. 2019 Dec 17;12(12):e002711. doi: 10.1161/CIRCGEN.119.002711 (PMC6922071; doi:10.1161/CIRCGEN.119.002711)
Supplement: Supplementary file 1 [file hcg-12-e002711-s001.pdf]

Supplemental Materials

FULL LIST OF INVENT CONSORTIUM MEMBERS

Sara Lindstrom, PhD,[1] Lu Wang, PhD,[2] Erin N. Smith, PhD,[3] William Gordon, MS,[4] Astrid van Hylckama Vlieg, PhD,[5] Mariza de Andrade, PhD,[6] Jennifer A. Brody, BA,[7] Jack W. Pattee, BA,[8] Jeffrey Haessler, MS,[9] Ben M. Brumpton, PhD, MPH,[10] Daniel I. Chasman, PhD,[11] Pierre Suchon, MD-PhD,[12] Ming-Huei Chen, PhD, [13] Constance Turman, MS,[14] Marine Germain,[15] Kerri L. Wiggins, MS, RD,[16] James MacDonald, MS,[17] Sigrid K. Braekkan, PhD,[18] Sebastian M. Armasu, MS,[19] Nathan Pankratz, PhD,[20] Rabecca D. Jackson, MD,[21] Jonas B. Nielsen, MD, PhD,[22] Franco Giulianini, PhD,[23] Marja K. Puurunen, MD, PhD,[24] Manal Ibrahim, MD,[25] Susan R. Heckbert, MD, PhD,[26] Scott M. Damrauer, MD,[27] Pradeep Natarajan, MD, MMSc,[28] Derek Klarin, MD,[29] The Million Veteran Program, Paul S. de Vries, PhD,[30] Maria Sabater-Lleal, PhD,[31] Jennifer E. Huffman, PhD,[32] The CHARGE Hemostasis Working Group, Theo K. Bammler, PhD,[33] Kelly A. Frazer, PhD,[34] Bryan M. McCauley, MS,[35] Kent Taylor, PhD,[36] James S. Pankow, PhD, MPH,[37] Alexander P. Reiner, MD, MPH,[38] Maiken E. Gabrielsen, PhD,[39] Jean-François Deleuze, PhD,[40] Chris J. O'Donnell, MD,[41] Jihye Kim, PhD, MPH,[42] Barbara McKnight, PhD,[43] Peter Kraft, PhD,[44] JohnBjarne Hansen, MD, PhD,[45] Frits R. Rosendaal, MD, PhD,[46] John A. Heit, MD,[47] Bruce M. Psaty, MD, PhD,[48] Weihong Tang, MD, PhD,[49] Charles Kooperberg, PhD,[50] Kristian Hveem, MD, PhD,[51] Paul M. Ridker, MD, MPH,[52] PierreEmmanuel Morange, MD-PhD,[53] Andrew D. Johnson, PhD,[54] Christopher Kabrhel, MD MPH,[55] David-AlexandreTrégouët, PhD,[56] Nicholas L. Smith, PhD,[57] on behalf of the INVENT Consortium

[1] Department of Epidemiology, University of Washington, Seattle, Washington, USA; Public Health Sciences Division, Fred Hutchinson Cancer Research Center, Seattle, WA, USA.
[2] Department of Environmental and Occupational Health Sciences, University of Washington, Seattle, Washington, USA.
[3] Department of Pediatrics and Rady Children's Hospital, University of California San Diego, La Jolla, USA; K.G. Jebsen Thrombosis Research and Expertise Center, Department of Clinical Medicine, UiT – The Arctic University of Norway, Tromsø, Norway.
[4] Department of Epidemiology, University of Washington, Seattle, WA, USA.
[5] Department of Clinical Epidemiology, Leiden University Medical Center, Leiden, The Netherlands.
[6] Department of Health Sciences Research, Mayo Clinic, Rochester, MN USA.
[7] Cardiovascular Health Research Unit, Department of Medicine, University of Washington, Seattle WA USA.
[8] Division of Biostatistics, School of Public Health, University of Minnesota, Minneapolis, MN USA.
[9] Division of Public Health, Fred Hutchinson Cancer Research Center, Seattle WA, USA.
[10] K.G. Jebsen Center for Genetic Epidemiology, Department of Public Health and Nursing, NTNU, Norwegian University of Science and Technology, Trondheim, Norway; MRC Integrative Epidemiology Unit, University of Bristol, Bristol, UK; Clinic of Thoracic and Occupational Medicine, St. Olavs Hospital, Trondheim University Hospital, Trondheim, Norway.
[11] Division of Preventive Medicine, Brigham and Women's Hospital, Boston, USA; Harvard Medical School, Boston, USA.
[12] Laboratory of Haematology, La Timone Hospital, Marseille, France; C2VN, Aix Marseille University, INSERM, INRA, C2VN, Marseille, France.
[13] Population Sciences Branch, Division of Intramural Research, National Heart, Lung and Blood Institute, Bethesda, MD, USA; NHLBI and Boston University's The Framingham Heart Study, Framingham, MA, USA.
[14] Program in Genetic Epidemiology and Statistical Genetics, Harvard T.H. Chan School of Public Health, Boston, MA, USA.
[15] INSERM UMR_S 1219, Bordeaux Population Health Research Center, University of Bordeaux, France.

[16] Cardiovascular Health Research Unit, Department of Medicine, University of Washington, Seattle WA USA.
[17] Department of Environmental and Occupational Health Sciences, University of Washington, Seattle, Washington, USA.
[18] K.G. Jebsen Thrombosis Research and Expertise Center, Department of Clinical Medicine, UiT – The Arctic University of Norway, Tromsø, Norway; Division of Internal Medicine, University Hospital of North Norway, Tromsø, Norway.
[19] Health Sciences Research, Mayo Clinic, Rochester, MN USA.
[20] Department of Laboratory Medicine and Pathology, School of Medicine, University of Minnesota, Minneapolis, MN, USA.
[21] Division of Endocrinology, Diabetes and Metabolism, The Ohio State University, Columbus OH, USA.
[22] Department of Internal Medicine, Division of Cardiology, University of Michigan Medical School, Ann Arbor, Michigan, USA.
[23] Division of Preventive Medicine, Brigham and Women's Hospital, Boston, USA.
[24] NHLBI and Boston University's The Framingham Heart Study, Framingham, MA, USA.
[25] Laboratory of Haematology, La Timone Hospital, Marseille, France.; C2VN, Aix Marseille University, INSERM, INRA, C2VN, Marseille, France.
[26] Department of Epidemiology, University of Washington, Seattle, Washington, USA; Kaiser Permanente Washington Health Research Institute, Kaiser Permanente Washington, Seattle WA USA.
[27] Department of Surgery, Corporal Michael Crescenz VA Medical Center, Philadelphia, USA; Department of Surgery, Perleman School of Medicine, University of Pennsylvania, Philadelphia, USA.
[28] Boston VA Healthcare System, Boston, MA, USA; Cardiovascular Research Center and Center for Genomic Medicine, Massachusetts General Hospital, Boston, MA, USA; Department of Medicine, Harvard Medical School, Boston, MA, USA; Program in Medical and Population Genetics, Broad Institute of Harvard and MIT, Cambridge, MA, USA.
[29] Boston VA Healthcare System, Boston, Massachusetts, USA; Center for Genomic Medicine, Massachusetts General Hospital, Harvard Medical School, Boston, Massachusetts, USA; Program in Medical and Population Genetics, Broad Institute of MIT and Harvard, Cambridge, Massachusetts, USA; Department of Surgery, Massachusetts General Hospital, Boston, Massachusetts, USA.
[30] Human Genetics Center, Department of Epidemiology, Human Genetics, and Environmental Sciences, School of Public Health, The University of Texas Health Science Center at Houston, Houston, TX, USA.
[31] Unit of Genomics of Complex Diseases, Institut de Recerca de l'Hospital de Sant Pau, IIB-Sant Pau, Barcelona, Spain; Cardiovascular Medicine Unit, Department of Medicine, Karolinska Institutet, Center for Molecular Medicine, Karolinska University Hospital, Stockholm, Sweden.
[32] Center for Population Genomics, MAVERIC, VA Boston Healthcare System, Boston, MA, USA.
[33] Department of Environmental and Occupational Health Sciences, University of Washington, Seattle, Washington, USA.
[34] Department of Pediatrics and Rady Children's Hospital, University of California San Diego, La Jolla, USA; K.G. Jebsen Thrombosis Research and Expertise Center, Department of Clinical Medicine, UiT – The Arctic University of Norway, Tromsø, Norway; Institute of Genomic Medicine, University of California San Diego, La Jolla, California, USA.
[35] Health Sciences Research, Mayo Clinic, Rochester, MN USA.
[36] Los Angeles Biomedical Research Institute and Department of Pediatrics, Harbor-UCLA Medical Center, Torrence CA 90502, USA.
[37] Division of Epidemiology and Community Health, School of Public Health, University of Minnesota, Minneapolis, Minnesota, USA.
[38] Department of Epidemiology, University of Washington, Seattle WA, United States; Division of Public Health, Fred Hutchinson Cancer Research Center, Seattle WA, United States.

[39] K.G. Jebsen Center for Genetic Epidemiology, Department of Public Health and Nursing, NTNU, Norwegian University of Science and Technology, Trondheim, Norway.
[40] Centre National de Recherche en Génomique Humaine, Direction de la Recherche Fondamentale, CEA, 91057 Evry, France; CEPH, Fondation Jean Dausset, Paris, France.
[41] Million Veteran's Program, Veteran's Administration, Boston, MA; Population Sciences Branch, Division of Intramural Research, National Heart, Lung and Blood Institute, Bethesda, MD, USA; NHLBI and Boston University's The Framingham Heart Study, Framingham, MA, USA.
[42] Program in Genetic Epidemiology and Statistical Genetics, Harvard T.H. Chan School of Public Health, Boston, MA, USA.
[43] Department of Biostatistics, University of Washington, Seattle WA USA; Division of Public Health Sciences, Fred Hutchinson Cancer Research Center, Seattle, WA, USA.
[44] Program in Genetic Epidemiology and Statistical Genetics, Harvard T.H. Chan School of Public Health, Boston, MA, USA.
[45] K.G. Jebsen Thrombosis Research and Expertise Center, Department of Clinical Medicine, UiT – The Arctic University of Norway, Tromsø, Norway; Division of Internal Medicine, University Hospital of North Norway, Tromsø, Norway.
[46] Department of Clinical Epidemiology, Leiden University Medical Center, Leiden, The Netherlands.
[47] Health Sciences Research, Mayo Clinic, Rochester, MN USA.
[48] Cardiovascular Health Research Unit, Departments of Medicine, Epidemiology, and Health Services, University of Washington, Seattle WA USA; Kaiser Permanente Washington Health Research Institute, Kaiser Permanente Washington, Seattle WA USA.
[49] Division of Epidemiology and Community Health, School of Public Health, University of Minnesota, Minneapolis, Minnesota, USA.
[50] Division of Public Health, Fred Hutchinson Cancer Research Center, Seattle WA, United States.
[51] K.G. Jebsen Center for Genetic Epidemiology, Department of Public Health and Nursing, NTNU, Norwegian University of Science and Technology, Trondheim, Norway.
[52] Division of Preventive Medicine, Brigham and Women's Hospital, Boston, USA; Harvard Medical School, Boston, USA.
[53] C2VN, Aix Marseille Univ, INSERM, INRA, C2VN, Marseille, France; Laboratory of Haematology, La Timone Hospital, Marseille, France; CRB Assistance Publique - Hôpitaux de Marseille, HemoVasc (CRB AP-HM HemoVasc), Marseille, France.
[54] Population Sciences Branch, Division of Intramural Research, National Heart, Lung and Blood Institute, Bethesda, MD, USA; NHLBI and Boston University's The Framingham Heart Study, Framingham, MA, USA.
[55] Center for Vascular Emergencies, Department of Emergency Medicine, Massachusetts General Hospital; Channing Division of Network Medicine, Brigham and Women's Hospital; Harvard Medical School.
[56] INSERM UMR_S 1219, Bordeaux Population Health Research Center, University of Bordeaux, France.
[57] Department of Epidemiology, University of Washington, Seattle, Washington, USA; Kaiser Permanente Washington Health Research Institute, Kaiser Permanente Washington, Seattle WA USA; Seattle Epidemiologic Research and Information Center, Department of Veterans Affairs Office of Research and Development, Seattle WA USA. On behalf of the INVENT Consortium.

SUPPLEMENTARY METHODS

Study design and data sources

We performed two-sample Mendelian randomization analyses, taking genetic associations with lipid concentrations from one dataset, and genetic associations with cardiovascular disease outcomes from an independent dataset. Two-sample Mendelian randomization was performed in preference to one-sample Mendelian randomization using lipid measurements in UK Biobank to avoid the possibility of false positive findings arising due to weak-instrument bias, which can occur in a one-sample analysis.[1]

We obtained genetic associations with lipid concentrations (LDL-cholesterol, HDL-cholesterol, and triglycerides) from the Global Lipids Genetics Consortium (GLGC) on up to 188,577 participants of European ancestries. [2, 3] We decided to use variants and associations from the 2013 version of GLGC results, and not the later exome release,[4] to make sure that both exonic and intronic variants were treated consistently in the analysis. Genetic associations were estimated with adjustment for age, sex, and genomic principal components within each participating study after inverse rank quantile normalization of lipid concentrations, and then meta-analysed across studies. Inverse rank quantile normalization means that associations are not affected by the skewed distribution of variables, in particular for triglycerides. The triglyceride measurement mostly represents the triglyceride content of triglyceride-rich lipoproteins.

We estimated genetic associations with disease outcomes from UK Biobank, a cohort of 502,682 participants (94% of self-reported European ancestries) recruited between 2006-2010 in 22 assessment centres throughout the UK and followed-up until 31st

March 2017 or their date of death (recorded until 14th February 2018).[5] In addition to standard genetic and individual-level quality control procedures (call rate ≥ 99%, info score > 0.9, Hardy-Weinberg equilibrium p-value ≥ 10^{-5}, removal of genetic sex mismatches),[6] we excluded participants having non-European ancestries (self-report or inferred by genetics) or excess heterozygosity (>3 standard deviations from the mean). We included only one of each set of related participants (third-degree relatives or closer). The sample for our analyses comprised 367,703 unrelated participants of European ancestries.

We defined 19 outcomes based on electronic health records (ICD-9 or ICD-10 diagnosis and hospital procedure codes) from hospital episode statistics and death certificates, and self-reported information validated by interview at baseline with a trained nurse. With respect to study baseline, we included both prevalent and incident events in our outcome definitions. However, with respect to the genetic variants, all events are incident. For presentation, we divide outcomes into five categories: 1) ischaemic cerebrovascular diseases (ischaemic stroke, transient ischaemic attack, combined ischaemic cerebrovascular event [i.e. ischaemic stroke or transient ischaemic attack]), 2) haemorrhagic stroke (intracerebral haemorrhage, subarachnoid haemorrhage, combined haemorrhagic stroke); 3) aneurysms (any aortic aneurysm, abdominal aortic aneurysm, thoracic aortic aneurysm); 4) thromboembolic diseases (any venous thromboembolism, pulmonary embolism, deep vein thrombosis); and 5) other cardiovascular diseases (hypertension, peripheral vascular disease, aortic valve stenosis, atrial fibrillation, and heart failure). Additionally, we included CAD as a positive control outcome and chronic kidney disease as a negative control outcome as it is thought to be influenced by some cardiovascular risk factors (such as hypertension and diabetes) but not dyslipidaemia.[7] Precise outcome definitions are given in

Supplementary Table S14. For composite outcomes (such as any venous thromboembolism), if an individual had both the constituent outcomes (here, deep vein thrombosis and pulmonary embolism), then they were included as a case in each analysis. To obtain genetic association estimates for each outcome, we conducted logistic regression with adjustment for age at recruitment, sex and 10 genomic principal components using the *snptest* program.[8] These summarized data are required to combine evidence across datasets in a two-sample Mendelian randomization analysis.

Exploratory analyses

To validate unexpected results in the primary Mendelian randomization analyses, we performed additional exploratory analyses for relevant continuous traits. To provide further evidence on the link between triglycerides and thromboembolism, we performed multivariable Mendelian randomization analyses with tissue-type plasminogen activator and platelet count as outcomes. Tissue-type plasminogen activator was chosen as it was associated with triglycerides in a previous observational study among 1227 men free of coronary heart disease.[9] Genetic associations with tissue-type plasminogen activator were obtained from a genome-wide association study (GWAS) of 83 proteins in 3,394 participants of European ancestries.[10] Platelet count was chosen as excess thrombocytopenia events were observed in the treatment arm of the APPROACH trial of the APOC3 inhibitor volanesorsen.[11] Genetic associations with platelet count were obtained from a GWAS of blood cell traits in up to 173,480 participants of European ancestries.[6] To provide further evidence on the links between HDL-cholesterol and triglycerides with hypertension, we performed multivariable Mendelian randomization analyses with systolic and diastolic blood

pressure as outcomes. Genetic associations with blood pressure were obtained from up to 757,601 European-ancestry participants.[12]

Statistical analysis

We carried out analyses based on 184 genetic variants previously demonstrated to be associated with at least one of LDL-cholesterol, HDL-cholesterol or triglycerides at a genome-wide level of significance ($p < 5 \times 10^{-8}$) in the GLGC. Only one variant per gene region was included in the analysis, except for a small number of regions where a conditionally independent signal was found. We accounted for correlations between variants in the analysis methods, except for the weighted median method, where we further pruned to only include one variant per gene region, and correlations were derived in 502 European descent participants from the 1000 Genomes Project Phase 3. These variants explained 13.7% of the variance in HDL-cholesterol, 14.6% in LDL-cholesterol, and 11.7% in triglycerides in GLGC. One variant (rs2954022) was omitted from analyses as it was triallelic in UK Biobank but all other variants were well-imputed (info score >0.9) and were retained.

To obtain the associations of genetically-predicted values of these lipids with each cardiovascular outcome while accounting for measured genetic pleiotropy via other major lipids, we performed multivariable Mendelian randomization analyses.[13] We implemented this method by weighted regression of the genetic associations with the outcome on the genetic associations with the three predictors, while fixing the intercept to be zero. For sensitivity analysis, we also performed (i) univariable Mendelian randomization using the inverse-variance weighted method for each lipid fraction based on all variants associated with that lipid fraction at a genome-wide level of significance,[14] (ii) MR-Egger regression to account for unmeasured pleiotropy,[15] and

(iii) weighted median regression to assess robustness to invalid genetic instruments.[16] To account for between-variant heterogeneity, we used multiplicative random-effects models in all analyses. Heterogeneity statistics for the study outcomes are reported in Supplementary Tables S2 and S3. All Mendelian randomization estimates are expressed per 1 standard deviation increase in the corresponding lipid fraction in GLGC (1 standard deviation was 39.0 mg/dL for LDL-cholesterol, 15.8 mg/dL for HDL-cholesterol, and 90.5 mg/dL for triglycerides).

For cardiovascular diseases for which it is possible that effect of lipid fractions on that outcome may act via other conditions (e.g. via CAD for heart failure), we also performed multivariable Mendelian randomization analyses adjusted for the hypothesised mediating condition. This analysis estimates the direct effects of the lipid fractions on the outcome that are not mediated via CAD. The coefficient for CAD represents the odds ratio for the outcome per unit increase in the beta-coefficient for CAD (i.e. per unit increase in log odds ratio for CAD) Additionally, we performed multivariable Mendelian randomization analyses for these outcomes excluding participants with a CAD diagnosis, to assess whether associations persisted in participants without comorbid CAD. To assess the effect of triglycerides on molecular traits related to thrombosis, we searched publicly-available genetic associations from PhenoScanner (http://www.phenoscanner.medschl.cam.ac.uk/),[17] a curated database of over 60 billion genetic associations, and performed multivariable Mendelian randomization using these traits as outcomes. Finally, we performed replication and meta-analysis of the most surprising finding of this study through collaborating with the INVENT consortium.[18] In this replication, we requested exclusion of non-European-ancestry participants and of participants from the UK

Biobank, and performed the same multivariable Mendelian randomization methods applied in the main analyses.

We also performed gene-specific analyses for variants in specific gene regions that can be considered as proxies for existing or proposed lipid-lowering therapies (since the protein products of these genes are the pharmacological targets of these drugs) and that explain enough variance in lipid concentration to result in adequately powered analyses (at least 0.4% for at least one lipid measure). We considered the following gene regions: *HMGCR* (proxy for statin treatment), *PCSK9* (proxy for PCSK9 inhibitors), *LDLR* (proxy for inhibition of the LDL receptor), *APOC3* (proxy for APOC3 inhibitors), and *LPL* (proxy for lipoprotein lipase inhibition). For each gene region, we selected genetic variants that were not strongly correlated ($R^2<0.8$) with one another and that were previously associated with LDL-cholesterol in a conditional analysis (Supplementary Table S15). All variants are within 500kb of the gene. We obtained univariable Mendelian randomization estimates using the inverse-variance weighted method while accounting for correlations between variants.[14] Variants in each gene region explained 0.4% (*HMGCR*), 1.2% (*PCSK9*), 1.0% (*LDLR*), 0.1% (*APOC3*) and less than 0.1% (*LPL*) of the variance in LDL-cholesterol in GLGC. The *APOC3* and *LPL* variants also explained 1.0% and 0.9% of the variance in triglycerides respectively. The power of the gene-specific analyses differs considerably owing to the different proportion of variance explained. Gene-specific estimates are expressed per 1 standard deviation increase in LDL-cholesterol for the *HMGCR*, *PCSK9*, and *LDLR* regions, and per 1 standard deviation increase in triglycerides for the *APOC3* and *LPL* regions.

We carried out all analyses using R (version 3.4.4) unless otherwise stated. All p-values presented are two-sided. To account for multiple testing, we use a Bonferroni-

corrected threshold of $p < 0.05/19 = 0.003$ for statistical significance. P-values between 0.003 and 0.05 are described as “nominally significant”.

SUPPLEMENTARY RESULTS

Supplementary Table S1: Estimates (odds ratio per 1 standard deviation increase in lipid fraction and 95% confidence interval) from polygenic multivariable Mendelian randomization analyses for all lipid-related variants. Estimates with $p < 0.05$ are reported in **bold**.

DISEASE	HDL-CHOLESTEROL	LDL-CHOLESTEROL	TRIGLYCERIDES
Coronary Artery Disease	**0.91 (0.83-1.00)**	**1.45 (1.35-1.57)**	**1.25 (1.12-1.40)**
Ischaemic Cerebrovascular Disease (all)	0.95 (0.86-1.05)	**1.14 (1.04-1.24)**	0.98 (0.87-1.10)
Ischaemic Stroke	0.98 (0.86-1.12)	1.10 (0.98-1.23)	1.03 (0.87-1.20)
Transient Ischaemic Attack	0.91 (0.80-1.03)	**1.16 (1.04-1.29)**	0.94 (0.81-1.10)
Haemorrhagic Stroke (all)	**0.76 (0.63-0.92)**	0.90 (0.76-1.05)	**0.78 (0.62-0.98)**
Intracerebral Haemorrhage	**0.65 (0.51-0.82)**	0.86 (0.70-1.05)	**0.65 (0.49-0.86)**
Subarachnoid Haemorrhage	0.82 (0.63-1.06)	0.90 (0.72-1.12)	0.85 (0.62-1.17)
Aortic Aneurysm (all)	**0.79 (0.65-0.96)**	**1.43 (1.21-1.68)**	1.06 (0.84-1.33)
Abdominal Aortic Aneurysm	**0.65 (0.51-0.85)**	**1.75 (1.40-2.17)**	0.94 (0.68-1.28)
Thoracic Aortic Aneurysm	0.81 (0.53-1.22)	0.98 (0.69-1.38)	0.93 (0.57-1.54)
Venous Thromboembolism (all)	0.94 (0.82-1.08)	1.03 (0.92-1.16)	**0.79 (0.67-0.93)**
Deep Vein Thrombosis	0.94 (0.81-1.09)	1.00 (0.88-1.13)	**0.78 (0.65-0.93)**
Pulmonary Embolism	0.98 (0.83-1.17)	1.12 (0.97-1.29)	**0.78 (0.64-0.96)**
Hypertension	**0.90 (0.83-0.97)**	1.05 (0.98-1.12)	**1.17 (1.07-1.27)**
Peripheral Vascular Disease	0.88 (0.75-1.03)	**1.15 (1.01-1.31)**	1.01 (0.84-1.22)
Aortic Valve Stenosis	0.98 (0.82-1.18)	**1.46 (1.25-1.70)**	**1.29 (1.04-1.61)**
Atrial Fibrillation	0.94 (0.87-1.03)	1.07 (1.00-1.15)	0.93 (0.84-1.02)
Heart Failure	0.97 (0.87-1.08)	**1.17 (1.06-1.28)**	**1.19 (1.04-1.37)**
Chronic Kidney Disease	0.90 (0.81-1.00)	0.93 (0.85-1.02)	0.98 (0.87-1.12)

Supplementary Table S2: Heterogeneity statistics for the main multivariable analyses.

DISEASE	Q STATISTIC	HETEROGENEITY P-VALUE	I^2
Coronary Artery Disease	621.3	<0.001	70.9
Ischaemic Cerebrovascular Disease (all)	234.0	0.005	22.7
Ischaemic Stroke	239.5	0.002	24.4
Transient Ischaemic Attack	187.1	0.362	3.3
Haemorrhagic Stroke	217.9	0.032	16.9
Intracerebral Haemorrhage	153.8	0.930	0.0
Subarachnoid Haemorrhage	219.6	0.027	17.6
Aortic Aneurysm (all)	203.9	0.117	11.2
Abdominal Aortic Aneurysm	218.8	0.029	17.3
Thoracic Aortic Aneurysm	164.1	0.812	0.0
Venous Thromboembolism (all)	766.8	<0.001	76.4
Deep Vein Thrombosis	614.0	<0.001	70.5
Pulmonary Embolism	527.5	<0.001	65.7
Hypertension	1255.6	<0.001	85.6
Peripheral Vascular Disease	246.5	0.001	26.6
Aortic Valve Stenosis	224.3	0.016	19.3
Atrial Fibrillation	320.8	<0.001	43.6
Heart Failure	252.3	<0.001	28.3
Chronic Kidney Disease	206.0	0.098	12.2

Supplementary Table S3: Heterogeneity statistics and p-values for the main multivariable analyses from calculated standard inverse-variance weighted method with unmodified weights, and calculated using MR-PRESSO package (bootstrapped p-values).

DISEASE	*STANDARD Q STATISTIC*	*P-VALUE*	*MR-PRESSO Q STATISTIC*	*MR-PRESSO P-VALUE*
Coronary Artery Disease	621.3	<0.001	651.7	<0.001
Ischaemic Cerebrovascular Disease (all)	234.0	0.005	240.8	0.008
Ischaemic Stroke	239.5	0.002	247.8	<0.001
Transient Ischaemic Attack	187.1	0.362	192.8	0.378
Haemorrhagic Stroke	217.9	0.032	224.8	0.029
Intracerebral Haemorrhage	153.8	0.930	157.4	0.937
Subarachnoid Haemorrhage	219.6	0.027	228.8	0.022
Aortic Aneurysm (all)	203.9	0.117	211.0	0.121
Abdominal Aortic Aneurysm	218.8	0.029	227.0	0.030
Thoracic Aortic Aneurysm	164.1	0.812	170.6	0.822
Venous Thromboembolism (all)	766.8	<0.001	805.7	<0.001
Deep Vein Thrombosis	614.0	<0.001	644.1	<0.001
Pulmonary Embolism	527.5	<0.001	552.5	<0.001
Hypertension	1255.6	<0.001	1290.3	<0.001
Peripheral Vascular Disease	246.5	0.001	254.8	0.003
Aortic Valve Stenosis	224.3	0.016	233.5	0.012
Atrial Fibrillation	320.8	<0.001	331.1	<0.001
Heart Failure	252.3	<0.001	261.7	<0.001
Chronic Kidney Disease	206.0	0.098	212.3	0.104

Supplementary Figure S1: Scatter plots of genetic associations with lipid fraction (risk factor) and cardiovascular disease (outcome) for selected risk factor/outcome pairs. Genetic associations with the risk factor are in standard deviation units. Genetic associations with the outcome are log odds ratios. The line through the origin represents the estimate from the relevant univariable inverse-variance weighted method.

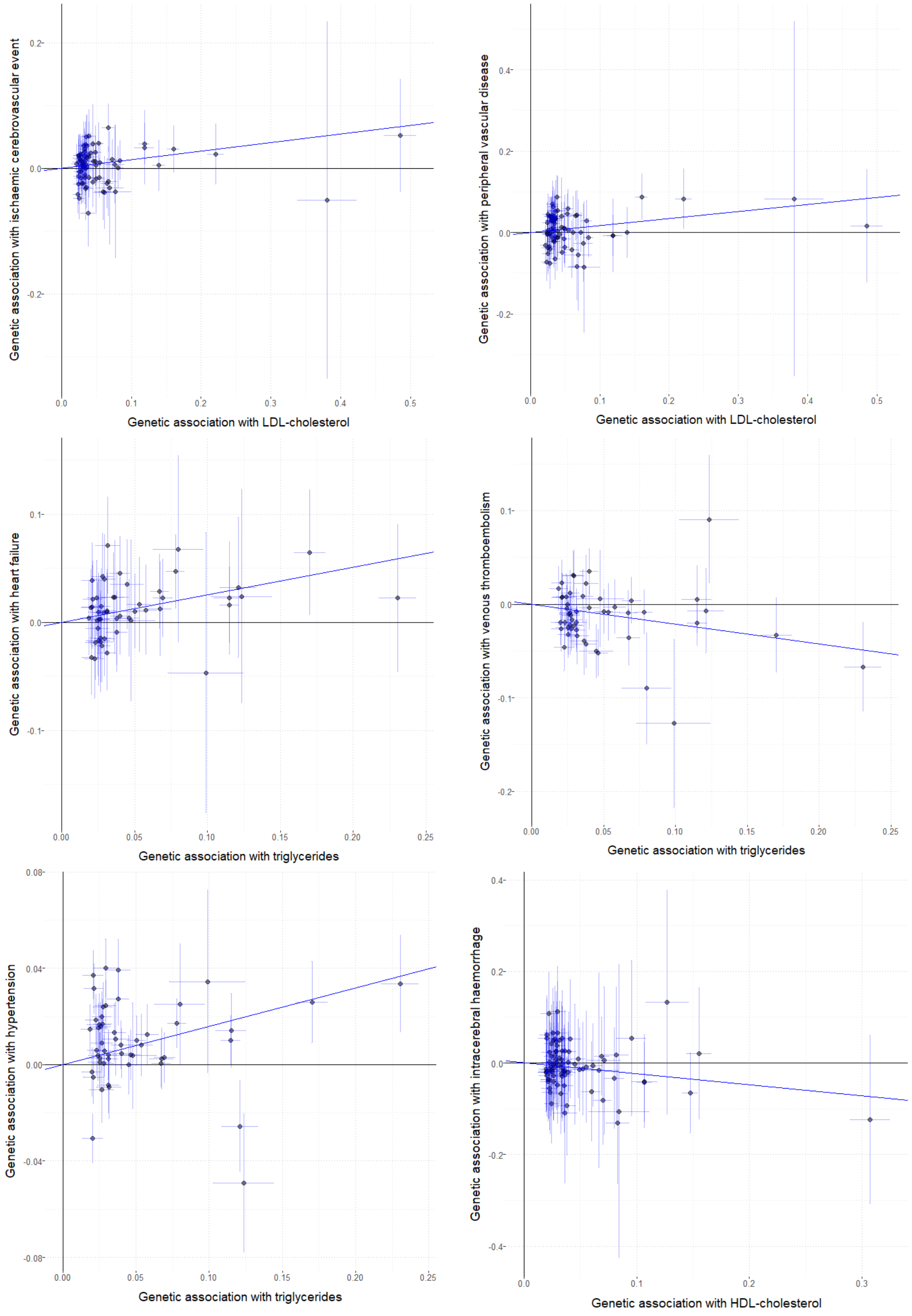

Supplementary Table S4: Associations of genetically-predicted triglyceride levels with traits relating to thrombosis.

TRAIT	STUDY	PARTICIPANTS	VARIANTS	BETA (95%CI)	P-VALUE
Tissue-type plasminogen activator	Folkersen 2017[10]	3,394	179	**0.19 (0.02 to 0.35)**	**0.031**
Platelet count	Astle 2016[6]	173,480	182	0.04 (-0.05 to 0.13)	0.407

Beta coefficients are scaled to a standard deviation (SD) change in the trait per 1 SD increase in genetically-predicted triglyceride levels. Estimates were obtained from multivariable Mendelian randomization accounting for genetic associations with LDL-cholesterol and HDL-cholesterol.

Supplementary Table S5: Replication of the associations between lipids and hypertension, using publicly available data on systolic and diastolic blood pressure in up to 757,601 participants of European ancestries[12]

	**HDL-cholesterol**	**LDL-cholesterol**	**Triglycerides**
Systolic-blood-pressure	-0.40 (-1.00 to 0.20)	0.20 (-0.31 to 0.71)	1.00 (0.27 to 1.73)*
Diastolic-blood-pressure	-0.19 (-0.62 to 0.23)	-0.21 (-0.57 to 0.15)	0.77 (0.26 to 1.29)*

*: $p < 0.05$ †: $p < 0.003$ ‡: $p < 10^{-8}$

Supplementary Figure S2: Estimates (odds ratio per 1 standard deviation increase in lipid fraction and 95% confidence interval) for HDL-cholesterol from genome-wide Mendelian randomization analyses for all lipid-related variants associated with the target lipid fraction.

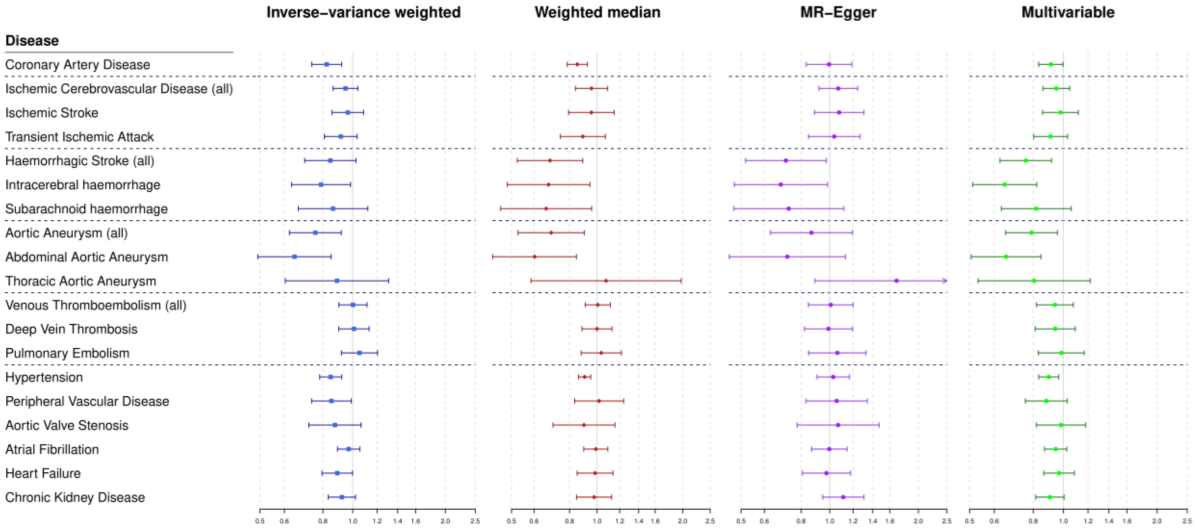

Supplementary Table S6: Estimates (odds ratio per 1 standard deviation increase in lipid fraction and 95% confidence interval) for HDL-cholesterol from polygenic univariable Mendelian randomization analyses for all lipid-related variants associated with the target lipid fraction. Estimates with $p < 0.05$ are reported in **bold**.

DISEASE	INVERSE-VARIANCE WEIGHTED	WEIGHTED MEDIAN	MR-EGGER REGRESSION
Coronary Artery Disease	**0.82 (0.74-0.92)**	**0.85 (0.78-0.92)**	0.99 (0.83-1.19)
Ischaemic Cerebrovascular Disease (all)	0.95 (0.86-1.04)	0.96 (0.84-1.09)	1.07 (0.92-1.24)
Ischaemic Stroke	0.96 (0.86-1.08)	0.95 (0.79-1.15)	1.08 (0.89-1.31)
Transient Ischaemic Attack	0.91 (0.81-1.03)	0.89 (0.74-1.07)	1.04 (0.85-1.27)
Haemorrhagic Stroke (all)	0.85 (0.70-1.03)	**0.68 (0.52-0.89)**	**0.71 (0.52-0.97)**
Intracerebral Haemorrhage	**0.79 (0.63-0.98)**	**0.68 (0.48-0.94)**	**0.68 (0.47-0.98)**
Subarachnoid Haemorrhage	0.86 (0.67-1.12)	**0.66 (0.46-0.96)**	0.73 (0.47-1.12)
Aortic Aneurysm (all)	**0.76 (0.62-0.92)**	**0.69 (0.53-0.90)**	0.87 (0.63-1.19)
Abdominal Aortic Aneurysm	**0.65 (0.49-0.85)**	**0.60 (0.43-0.85)**	0.72 (0.46-1.13)
Thoracic Aortic Aneurysm	0.89 (0.60-1.31)	**1.08 (0.59-1.98)**	1.69 (0.89-3.19)
Venous Thromboembolism (all)	1.00 (0.90-1.11)	1.01 (0.91-1.11)	1.01 (0.85-1.20)
Deep Vein Thrombosis	1.01 (0.90-1.13)	1.00 (0.88-1.13)	0.99 (0.82-1.20)
Pulmonary Embolism	1.05 (0.92-1.20)	1.03 (0.88-1.22)	1.06 (0.85-1.33)
Hypertension	**0.85 (0.78-0.92)**	**0.90 (0.86-0.95)**	1.03 (0.91-1.17)
Peripheral Vascular Disease	**0.85 (0.74-0.99)**	1.02 (0.83-1.24)	1.06 (0.83-1.34)
Aortic Valve Stenosis	0.88 (0.72-1.06)	0.90 (0.70-1.16)	1.07 (0.77-1.47)
Atrial Fibrillation	0.97 (0.89-1.05)	0.99 (0.90-1.09)	1.00 (0.87-1.15)
Heart Failure	**0.89 (0.79-1.00)**	0.98 (0.85-1.14)	0.97 (0.81-1.18)
Chronic Kidney Disease	0.92 (0.83-1.02)	0.98 (0.85-1.13)	1.11 (0.95-1.31)

Supplementary Figure S3: Estimates (odds ratio per 1 standard deviation increase in lipid fraction and 95% confidence interval) for LDL-cholesterol from genome-wide Mendelian randomization analyses for all lipid-related variants associated with the target lipid fraction.

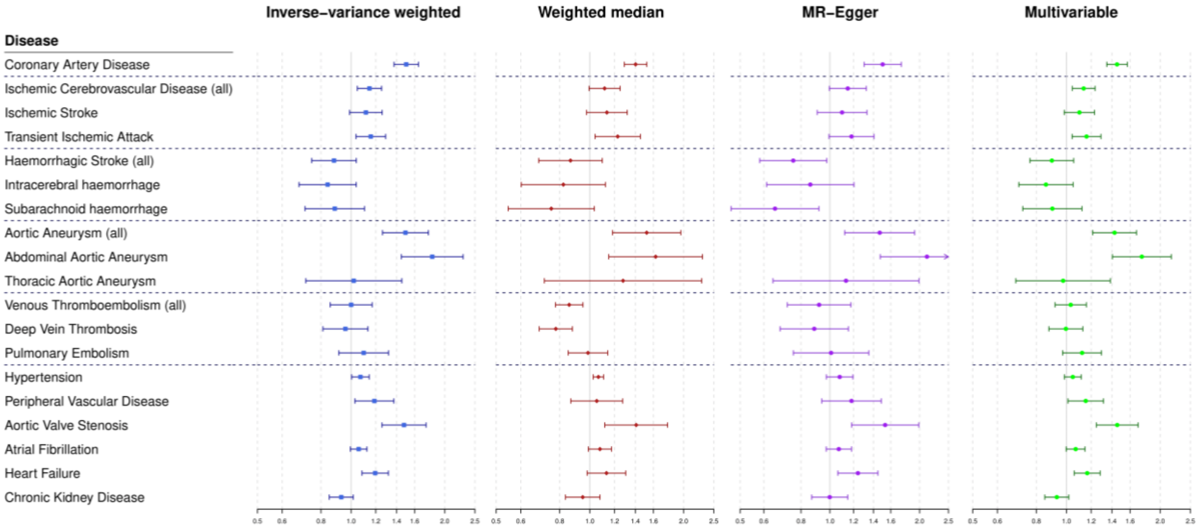

Supplementary Table S7: Estimates (odds ratio per 1 standard deviation increase in lipid fraction and 95% confidence interval) for LDL-cholesterol from polygenic univariable Mendelian randomization analyses for all lipid-related variants associated with the target lipid fraction. Estimates with $p < 0.05$ are reported in **bold**.

DISEASE	INVERSE-VARIANCE WEIGHTED	WEIGHTED MEDIAN	MR-EGGER REGRESSION
Coronary Artery Disease	**1.50 (1.37-1.65)**	**1.40 (1.29-1.52)**	**1.50 (1.30-1.74)**
Ischaemic Cerebrovascular Disease (all)	**1.15 (1.05-1.25)**	1.12 (0.99-1.25)	1.15 (1.00-1.32)
Ischaemic Stroke	1.12 (0.99-1.26)	1.13 (0.98-1.32)	1.10 (0.91-1.33)
Transient Ischaemic Attack	**1.16 (1.04-1.29)**	**1.23 (1.04-1.45)**	1.18 (0.99-1.41)
Haemorrhagic Stroke (all)	0.88 (0.75-1.04)	0.87 (0.69-1.10)	**0.75 (0.58-0.98)**
Intracerebral Haemorrhage	0.84 (0.68-1.04)	0.82 (0.60-1.12)	0.86 (0.61-1.20)
Subarachnoid Haemorrhage	0.89 (0.71-1.10)	0.75 (0.55-1.03)	**0.65 (0.47-0.92)**
Aortic Aneurysm (all)	**1.50 (1.26-1.77)**	**1.52 (1.18-1.96)**	**1.47 (1.12-1.92)**
Abdominal Aortic Aneurysm	**1.82 (1.45-2.29)**	**1.63 (1.15-2.30)**	**2.12 (1.48-3.04)**
Thoracic Aortic Aneurysm	1.02 (0.71-1.46)	1.28 (0.71-2.29)	1.13 (0.64-1.99)
Venous Thromboembolism (all)	1.00 (0.86-1.17)	**0.86 (0.78-0.95)**	0.92 (0.72-1.18)
Deep Vein Thrombosis	0.96 (0.81-1.13)	**0.78 (0.69-0.88)**	0.89 (0.68-1.15)
Pulmonary Embolism	1.10 (0.91-1.32)	0.99 (0.85-1.14)	1.01 (0.75-1.35)
Hypertension	**1.07 (1.00-1.14)**	**1.07 (1.03-1.11)**	1.08 (0.97-1.20)
Peripheral Vascular Disease	**1.19 (1.03-1.37)**	1.05 (0.87-1.27)	1.18 (0.94-1.49)
Aortic Valve Stenosis	**1.48 (1.26-1.74)**	**1.41 (1.12-1.78)**	**1.53 (1.18-1.99)**
Atrial Fibrillation	1.06 (0.99-1.12)	1.08 (0.99-1.17)	1.07 (0.97-1.18)
Heart Failure	**1.19 (1.08-1.32)**	1.13 (0.98-1.30)	**1.24 (1.06-1.45)**
Chronic Kidney Disease	0.93 (0.85-1.02)	0.95 (0.84-1.08)	1.00 (0.87-1.15)

Supplementary Figure S4: Estimates (odds ratio per 1 standard deviation increase in lipid fraction and 95% confidence interval) for triglycerides from genome-wide Mendelian randomization analyses for all lipid-related variants associated with the target lipid fraction.

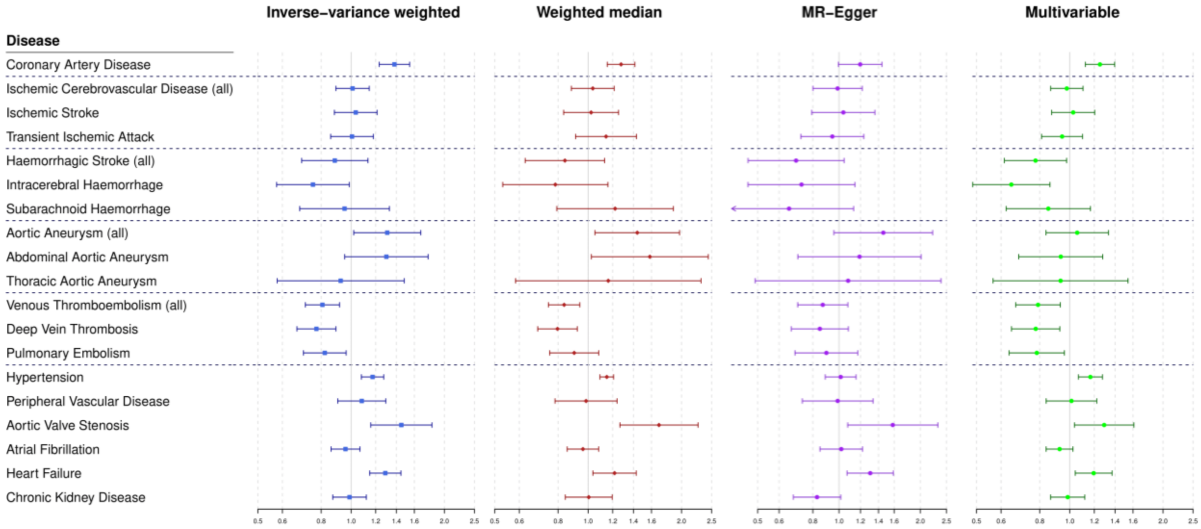

Supplementary Table S8: Estimates (odds ratio per 1 standard deviation increase in lipid fraction and 95% confidence interval) for triglycerides from polygenic univariable Mendelian randomization analyses for all lipid-related variants associated with the target lipid fraction. Estimates with $p < 0.05$ are reported in **bold**.

DISEASE	INVERSE-VARIANCE WEIGHTED	WEIGHTED MEDIAN	MR-EGGER REGRESSION
Coronary Artery Disease	**1.38 (1.23-1.54)**	**1.28 (1.15-1.41)**	1.20 (0.99-1.44)
Ischaemic Cerebrovascular Disease (all)	1.01 (0.89-1.14)	1.04 (0.88-1.21)	0.99 (0.80-1.22)
Ischaemic Stroke	1.03 (0.88-1.21)	1.02 (0.83-1.25)	1.04 (0.79-1.36)
Transient Ischaemic Attack	1.01 (0.86-1.18)	1.14 (0.91-1.43)	0.94 (0.72-1.24)
Haemorrhagic Stroke (all)	0.89 (0.69-1.13)	0.84 (0.63-1.13)	0.69 (0.46-1.04)
Intracerebral Haemorrhage	**0.75 (0.57-0.99)**	0.78 (0.53-1.16)	0.73 (0.46-1.15)
Subarachnoid Haemorrhage	0.95 (0.68-1.33)	1.22 (0.79-1.88)	0.65 (0.38-1.13)
Aortic Aneurysm (all)	**1.31 (1.02-1.68)**	**1.44 (1.05-1.97)**	1.46 (0.96-2.22)
Abdominal Aortic Aneurysm	1.30 (0.95-1.77)	**1.58 (1.02-2.44)**	1.19 (0.70-2.01)
Thoracic Aortic Aneurysm	0.93 (0.58-1.48)	1.16 (0.58-2.31)	1.08 (0.49-2.39)
Venous Thromboembolism (all)	**0.81 (0.71-0.92)**	**0.84 (0.74-0.94)**	0.87 (0.70-1.08)
Deep Vein Thrombosis	**0.77 (0.67-0.89)**	**0.80 (0.69-0.92)**	0.85 (0.67-1.08)
Pulmonary Embolism	**0.82 (0.70-0.96)**	0.90 (0.75-1.08)	0.90 (0.69-1.17)
Hypertension	**1.17 (1.08-1.27)**	**1.15 (1.09-1.21)**	1.01 (0.89-1.16)
Peripheral Vascular Disease	1.08 (0.91-1.29)	0.98 (0.78-1.24)	0.99 (0.73-1.34)
Aortic Valve Stenosis	**1.45 (1.16-1.82)**	**1.69 (1.27-2.26)**	**1.58 (1.08-2.32)**
Atrial Fibrillation	0.96 (0.86-1.07)	0.96 (0.86-1.08)	1.02 (0.85-1.22)
Heart Failure	**1.29 (1.15-1.45)**	**1.22 (1.04-1.43)**	**1.30 (1.07-1.59)**
Chronic Kidney Disease	0.99 (0.87-1.12)	1.00 (0.84-1.20)	0.83 (0.68-1.01)

Supplementary Table S9: Estimates (odds ratio per 1 standard deviation increase in lipid fraction and 95% confidence interval) from polygenic multivariable Mendelian randomization analyses for all lipid-related variants for heart failure in participants without a CAD diagnosis.

	**HDL-cholesterol**	**LDL-cholesterol**	**Triglycerides**
Heart Failure	1.04 (0.88-1.23)	0.87 (0.75-1.00)	1.12 (0.91-1.37)

Supplementary Table S10: Multivariable Mendelian randomization estimates (odds ratio with 95% confidence interval) for hypertension, abdominal aortic aneurysm and haemorrhagic stroke without and with adjustment for body mass index (BMI) and type 2 diabetes (T2D), and for venous thromboembolism with and without adjustment for heart failure (HF).

Hypertension	**HDL-cholesterol**	**LDL-cholesterol**	**Triglycerides**	**BMI/T2D**
Without adjustment	0.90 (0.83-0.97)*	1.05 (0.98-1.12)	1.17 (1.07-1.27)*	-
Adjustment for BMI	0.91 (0.84-0.98)*	1.06 (0.99-1.13)	1.17 (1.07-1.28)*	1.334 (1.08, 1.64)*
Adjustment for T2D	0.95 (0.89-1.02)	1.07 (1.01-1.13)*	1.15 (1.06-1.25)*	1.30 (1.19-1.40)†

Abdominal aortic aneurysm	**HDL-cholesterol**	**LDL-cholesterol**	**Triglycerides**	**T2D/BMI**
Without adjustment	0.65 (0.51-0.85)†	1.75 (1.40-2.17)†	0.94 (0.68-1.28)	-
Adjustment for BMI	0.64 (0.50-0.83)†	1.71 (1.38-2.13)†	0.93 (0.68-1.27)	0.57 (0.27, 1.21)
Adjustment for T2D	0.68 (0.52-0.89)†	1.77 (1.42-2.20)†	0.93 (0.68-1.27)	1.18 (0.86-1.61)

Haemorrhagic stroke	**HDL-cholesterol**	**LDL-cholesterol**	**Triglycerides**	**T2D/BMI**
Without adjustment	0.76 (0.63-0.92)†	0.90 (0.76-1.05)	0.78 (0.62-0.98)*	
Adjustment for BMI	0.76 (0.63-0.92)†	0.90 (0.76-1.06)	0.77 (0.61-0.98)*	0.98 (0.56, 1.70)
Adjustment for T2D	0.74 (0.61-0.90)†	0.89 (0.76-1.05)	0.78 (0.62-0.98)*	0.91 (0.73-1.15)

Venous thromboembolism	**HDL-cholesterol**	**LDL-cholesterol**	**Triglycerides**	**HF**
Without adjustment	0.94 (0.82-1.08)	1.03 (0.92-1.16)	0.79 (0.67-0.93)*	
Adjustment for HF	0.96 (0.85 to 1.09)	0.97 (0.87 to 1.09)	0.74 (0.63 to 0.87)†	1.45 (1.22 to 1.71)†

*: $p < 0.05$ †: $p < 0.003$ ‡: $p < 10^{-8}$

The BMI/T2D column indicates the odds ratio representing the association between genetically-predicted body mass index (per SD increase) or type 2 diabetes (per unit increase in log odds ratio) and the outcomes of interest (i.e. hypertension, abdominal aortic aneurysm, or haemorrhagic stroke) while accounting for the genetic associations of the lipid fractions in a multivariable analysis. If the regression coefficients for the lipid measurements remain unchanged on adjustment for BMI/T2D, then the effects of lipids on the outcome do not operate via BMI/T2D. If the regression coefficients attenuate to the null, then the effects of lipids on the outcome are entirely mediated via BMI/T2D.

A similar interpretation applies for the HF column for venous thromboembolism, which indicates the odds ratio between heart failure (per unit increase in log odds ratio) and venous thromboembolism while accounting for the genetic effects of circulating lipids on venous thromboembolism. This analysis shows that heart failure is confirmed as an independent risk factor for venous thromboembolism, but does not appear to mediate the effect of triglycerides on venous thromboembolism.

Supplementary Table S11: Estimates (odds ratio per 1 standard deviation increase in lipid fraction and 95% confidence interval) from gene-specific Mendelian randomization analyses for four specified gene regions. Estimates with $p < 0.05$ are reported in **bold**. Estimates are calibrated per standard deviation increase in LDL-cholesterol for *HMGCR*, *PCSK9*, and *LDLR* regions, and per standard deviation increase in triglycerides for *APOC3* and *LPL* regions.

DISEASE	*HMGCR*	*PCSK9*	*LDLR*	*APOC3*	*LPL*
Coronary Artery Disease	**1.35 (1.08-1.69)**	**1.32 (1.12-1.56)**	**1.58 (1.41-1.76)**	**1.27 (1.09-1.47)**	**1.65 (1.41-1.93)**
Ischaemic Cerebrovascular Disease (all)	1.15 (0.77-1.73)	1.03 (0.76-1.38)	1.05 (0.86-1.28)	1.00 (0.76-1.30)	**1.52 (1.21-1.92)**
Ischaemic Stroke	0.88 (0.52-1.50)	0.94 (0.64-1.39)	1.06 (0.81-1.38)	1.27 (0.89-1.80)	**1.45 (1.07-1.96)**
Transient Ischaemic Attack	1.33 (0.75-2.36)	1.02 (0.67-1.55)	1.07 (0.81-1.42)	0.79 (0.54-1.16)	**1.47 (1.06-2.04)**
Haemorrhagic Stroke	0.48 (0.22-1.08)	0.91 (0.51-1.65)	0.72 (0.49-1.08)	0.69 (0.41-1.18)	0.83 (0.52-1.32)
Intracerebral Haemorrhage	**0.28 (0.09-0.83)**	0.97 (0.43-2.16)	0.86 (0.50-1.48)	0.74 (0.36-1.52)	1.06 (0.56-1.98)
Subarachnoid Haemorrhage	0.72 (0.24-2.13)	0.99 (0.45-2.20)	**0.57 (0.33-0.98)**	0.55 (0.27-1.13)	0.61 (0.33-1.14)
Aortic Aneurysm (all)	1.43 (0.62-3.30)	1.19 (0.65-2.20)	**1.92 (1.27-2.89)**	**2.04 (1.17-3.53)**	**2.29 (1.22-4.29)**
Abdominal Aortic Aneurysm	2.73 (0.92-8.11)	1.64 (0.74-3.64)	**3.09 (1.81-5.28)**	1.70 (0.83-3.48)	**2.69 (1.44-5.00)**
Thoracic Aortic Aneurysm	0.56 (0.08-3.80)	0.50 (0.12-2.05)	0.96 (0.37-2.48)	1.98 (0.56-6.98)	1.82 (0.61-5.46)
Venous Thromboembolism (all)	1.13 (0.83-1.53)	0.84 (0.67-1.05)	**1.20 (1.04-1.40)**	**0.76 (0.62-0.92)**	0.86 (0.72-1.02)
Deep Vein Thrombosis	1.12 (0.77-1.63)	**0.72 (0.55-0.95)**	**1.20 (1.00-1.45)**	**0.80 (0.63-1.02)**	0.89 (0.72-1.10)
Pulmonary Embolism	1.18 (0.74-1.87)	1.08 (0.77-1.51)	**1.28 (1.02-1.60)**	**0.71 (0.53-0.97)**	0.80 (0.61-1.04)
Hypertension	1.04 (0.91-1.18)	0.97 (0.88-1.07)	**1.07 (1.00-1.14)**	**1.16 (1.07-1.27)**	**1.18 (1.09-1.27)**
Peripheral Vascular Disease	0.98 (0.53-1.83)	1.05 (0.67-1.65)	**1.61 (1.19-2.18)**	0.87 (0.58-1.30)	**1.66 (1.12-2.48)**
Aortic Valve Stenosis	0.90 (0.42-1.92)	1.39 (0.80-2.43)	**1.77 (1.22-2.58)**	1.62 (0.99-2.67)	**1.86 (1.18-2.93)**
Atrial Fibrillation	0.89 (0.67-1.18)	1.04 (0.85-1.29)	1.07 (0.93-1.24)	0.90 (0.75-1.09)	**1.25 (1.06-1.47)**
Heart Failure	1.17 (0.75-1.82)	1.10 (0.80-1.53)	**1.32 (1.06-1.64)**	1.11 (0.83-1.49)	**1.51 (1.11-2.05)**
Chronic Kidney Disease	0.77 (0.49-1.22)	1.06 (0.76-1.49)	1.06 (0.85-1.33)	1.09 (0.81-1.47)	1.19 (0.92-1.55)

Supplementary Table S12: P-values from gene-specific Mendelian randomization analyses for five specified gene regions.

DISEASE	*HMGCR*	*PCSK9*	*LDLR*	*APOC3*	*LPL*
Coronary Artery Disease	0.010	<0.001	<0.001	0.002	<0.001
Ischaemic Cerebrovascular Disease (all)	0.494	0.867	0.646	0.976	<0.001
Ischaemic Stroke	0.638	0.767	0.672	0.187	0.018
Transient Ischaemic Attack	0.331	0.935	0.620	0.231	0.021
Haemorrhagic Stroke	0.076	0.762	0.109	0.175	0.427
Intracerebral Haemorrhage	0.022	0.934	0.596	0.415	0.864
Subarachnoid Haemorrhage	0.547	0.983	0.04	0.104	0.120
Aortic Aneurysm (all)	0.403	0.574	0.002	0.011	0.010
Abdominal Aortic Aneurysm	0.070	0.223	<0.001	0.144	0.002
Thoracic Aortic Aneurysm	0.550	0.338	0.938	0.290	0.284
Venous Thromboembolism (all)	0.447	0.121	0.016	0.007	0.089
Deep Vein Thrombosis	0.539	0.019	0.046	0.078	0.278
Pulmonary Embolism	0.490	0.669	0.034	0.029	0.092
Hypertension	0.569	0.507	0.044	<0.001	<0.001
Peripheral Vascular Disease	0.961	0.834	0.002	0.490	0.012
Aortic Valve Stenosis	0.782	0.244	0.003	0.057	0.008
Atrial Fibrillation	0.409	0.690	0.324	0.295	0.008
Heart Failure	0.495	0.553	0.013	0.479	0.009
Chronic Kidney Disease	0.271	0.719	0.597	0.586	0.189

Supplementary Table S13: Comparison of main findings from this study with evidence from external genetic studies and clinical trials

OUTCOME	LIPID	NEW FINDING OR BROAD REPLICATION OF PREVIOUS GENETIC EVIDENCE (PMID OF PREVIOUS MR STUDIES)	BROAD AGREEMENT OR DISAGREEMENT WITH EVIDENCE FROM MAJOR RANDOMIZED TRIALS OR CLINICAL GUIDELINES OF LIPID-LOWERING MEDICATIONS (PMID)
Coronary Artery Disease	***LDL-C***	Replication (24474739)	Agreement (statins 22607822, PCSK9i 30403574)
	TG		Agreement (EPA 30415628)
Ischaemic Cerebrovascular Event	***LDL-C***	Replication (29535274)	Agreement (statins 24788967)
Intracerebral Haemorrhage	***HDL-C***	New	NA
Abdominal Aortic Aneurysm	***LDL-C***	Replication (29188294)	Agreement (various interventions including statin therapy 28859943)
	HDL-C		NA
Venous Thromboembolism	***TG***	New	NA
Hypertension	***TG***	New	NA
Peripheral Vascular Disease	***LDL-C***	New	Agreement (statins 27851991 and 28886620)
Aortic Valve Stenosis	***LDL-C***	Replication (25344734)	Disagreement (statins 26828749) – see Discussion for potential reasons
	TG		NA
Heart Failure	***LDL-C***	New	Agreement (statins 23747642 and 27206819)
	TG		NA

PMID, Pubmed study identifier. LDL-C, low-density lipoprotein. TG, triglycerides.

PCSK9i, proprotein convertase subtilisin-kexin type 9 ihibitor.

EPA, eicosapentaenoic acid. NA, not available.

In the interest of clarity, this table includes only findings that were at least nominally significant in the main multivariable Mendelian randomization analyses and were confirmed in subsequent sensitivity analyses.

Supplementary Table S14: Summary of disease outcomes considered: sources of information

OUTCOME	NUMBER OF CASES	ICD-9 DIAGNOSIS	ICD-10 DIAGNOSIS	OPCS PROCEDURE	SELF-REPORT*
Coronary Artery Disease	29,278	410.X, 411.X, 412.X, 414.0, 414.8, 414.9	I21.X, I22.X, I23.X, I24.X, I25.1, I25.2, I25.5, I25.6, I25.8, I25.9	K40.X, K41.X, K42.X, K43.X, K44.X, K45.X, K46.X, K49.X, K50.1, K50.2, K50.4, K75.X	Non-cancer illness code (20002), Surgical operation code (20004), Health condition diagnosed by doctor (6150)
Ischaemic Cerebrovascular Disease (all)	8084	433.X, 434.X, 435.X	G45.X, I63.X		Non-cancer illness code (20002)
Ischaemic Stroke	4602	433.X, 434.X	I63.X		Non-cancer illness code (20002)
Transient Ischaemic Attack	3962	435.X	G45.X		Non-cancer illness code (20002)
Haemorrhagic Stroke (all)	1981	430.X, 431.X	I60.X, I61.X		Non-cancer illness code (20002)
Intracerebral Haemorrhage	1064	431.X	I61.X		Non-cancer illness code (20002)
Subarachnoid Haemorrhage	1084	430.X	I60.X		Non-cancer illness code (20002)
Aortic Aneurysm (all)	1849	441.X	I71.X	L19.4, L19.5	Non-cancer illness code (20002), Surgical operation code (20004)
Abdominal Aortic Aneurysm	1094	441.3, 441.4	I71.3, I71.4	L19.4, L19.5	Non-cancer illness code (20002)
Thoracic Aortic Aneurysm	347	441.1, 441.2	I71.1, I71.2		Non-cancer illness code (20002)
Venous Thromboembolism (all)	14,097	415.1, 451.1, 452.X, 453.0, 453.4, 453.9,	I26.X, I80.1, I80.2, I81.X, I82.0	L90.2	Non-cancer illness code (20002), Health condition diagnosed by doctor (6152)
Deep Vein Thrombosis	9454	451.1	I80.2	L90.2	Non-cancer illness code (20002), Health condition diagnosed by doctor (6152)
Pulmonary Embolism	6148	415.1	I26.X		Non-cancer illness code (20002), Health condition diagnosed by doctor (6152)
Hypertension	125,846	401.X	I10		Non-cancer illness code (20002), Health condition diagnosed by doctor (6150), Medication for health condition (6177)
Peripheral Vascular Disease	3415	443.8, 443.9	I73.8, I73.9		Non-cancer illness code (20002)
Aortic Valve Stenosis	2244		I35.0, I35.2		Non-cancer illness code (20002)
Atrial Fibrillation	16,945	427.3	I48		Non-cancer illness code (20002)
Heart Failure	6712	402.01, 402.11, 402.91, 404.01, 404.11, 404.91, 404.03, 404.13, 404.93, 428.X	I11.0, I13.0, I13.2, I50.X		Non-cancer illness code (20002)
Chronic Kidney Disease	6321	585.X	N18.X		Non-cancer illness code (20002)

Note that .X means that all sub-codes are matched. Abbreviations: ICD = International Classification of Disease; OPCS = Office of Population Censuses and Surveys Classification of Surgical Operations and Procedures; TIA = transient ischaemic attack

* Health condition diagnosed by doctor (6150/6152) and Medication for health condition (6177) were self-reported from touchscreen; Non-cancer illness code (20002) and Surgical operation code (20004) were self-reported from interview with trained nurse.

Supplementary Table S15: Genetic variants included in gene-specific analyses for each region.

GENE REGION	SNP	POS (HG19)	EFFECT ALLELE	OTHER ALLELE	EFFECT ALLELE FREQUENCY	BETA	SE	P-VALUE
HMGCR	rs2006760	chr5:74562029	G	C	0.229	0.053	0.008	1.7×10^{-13}
HMGCR	rs2303152	chr5: 74641707	G	A	0.890	-0.042	0.006	1.1×10^{-9}
HMGCR	rs17238484	chr5:74648496	G	T	0.763	-0.063	0.006	1.4×10^{-21}
HMGCR	rs12916	chr5:74656539	C	T	0.412	0.073	0.004	7.8×10^{-78}
HMGCR	rs10066707	chr5: 74560579	A	G	0.396	0.050	0.005	3.0×10^{-19}
HMGCR	rs5909	chr5: 74656175	A	G	0.100	0.062	0.009	5.0×10^{-13}
PCSK9	rs2479394	chr1:55486064	G	A	0.281	0.039	0.004	1.6×10^{-19}
PCSK9	rs11206510	chr1:55496039	C	T	0.172	-0.083	0.005	2.4×10^{-53}
PCSK9	rs2149041	chr1:55502137	C	G	0.823	-0.064	0.005	1.4×10^{-35}
PCSK9	rs10888897	chr1:55513061	C	T	0.680	-0.064	0.004	2.5×10^{-50}
PCSK9	rs7552841	chr1:55518752	C	T	0.596	0.051	0.004	8.4×10^{-31}
PCSK9	rs562556	chr1:55524237	A	G	0.631	-0.037	0.004	5.4×10^{-15}
LDLR	rs6511720	chr19:11202306	G	T	0.902	0.221	0.006	3.9×10^{-262}
LDLR	rs688	chr19:11227602	C	T	0.553	-0.054	0.004	1.0×10^{-43}
APOC3	rs10790162	chr11:116639104	A	G	0.091	0.231	0.006	1.1×10^{-249}
APOC3	rs603446	chr11:116654435	C	T	0.553	0.050	0.003	3.9×10^{-43}
LPL	rs1801177	chr8:19805708	A	G	0.011	0.164	0.023	1.1×10^{-9}
LPL	rs268	chr8:19813529	A	G	0.986	-0.280	0.035	2.2×10^{-16}
LPL	rs301	chr8:19816934	C	T	0.459	-0.109	0.004	1.9×10^{-167}
LPL	rs326	chr8:19819439	A	G	0.673	0.087	0.005	1.0×10^{-63}
LPL	rs328	chr8:19819724	C	G	0.870	0.167	0.006	2.0×10^{-179}

Genetic associations (beta and standard error [SE]) are with LDL-cholesterol for the *HMGCR*, *PCSK9*, and *LDLR* gene regions, and with triglycerides for the *APOC3* and *LPL* gene regions. They are given in standard deviation units and are taken from the Global Lipids Genetics Consortium (see http://csg.sph.umich.edu/willer/public/lipids2013/).

SUPPLEMENTARY REFERENCES

1. Pierce BL and Burgess S. Efficient design for Mendelian randomization studies: subsample and 2-sample instrumental variable estimators. *American Journal of Epidemiology*. 2013;178:1177-1184.
2. Do R, Willer CJ, Schmidt EM, Sengupta S, Gao C, Peloso GM, Gustafsson S, Kanoni S, Ganna A, Chen J, et al. Common variants associated with plasma triglycerides and risk for coronary artery disease. *Nat Genet*. 2013;45:1345-52.
3. Willer CJ, Schmidt EM, Sengupta S, Peloso GM, Gustafsson S, Kanoni S, Ganna A, Chen J, Buchkovich ML, Mora S, et al. Discovery and refinement of loci associated with lipid levels. *Nat Genet*. 2013;45:1274-1283.
4. Liu DJ, Peloso GM, Yu H, Butterworth AS, Wang X, Mahajan A, Saleheen D, Emdin C, Alam D, Alves AC, et al. Exome-wide association study of plasma lipids in >300,000 individuals. *Nat Genet*. 2017;49:1758-1766.
5. Sudlow C, Gallacher J, Allen N, Beral V, Burton P, Danesh J, Downey P, Elliott P, Green J, Landray M, et al. UK biobank: an open access resource for identifying the causes of a wide range of complex diseases of middle and old age. *PLoS Med*. 2015;12:e1001779.
6. Astle WJ, Elding H, Jiang T, Allen D, Ruklisa D, Mann AL, Mead D, Bouman H, Riveros-Mckay F, Kostadima MA, et al. The Allelic Landscape of Human Blood Cell Trait Variation and Links to Common Complex Disease. *Cell*. 2016;167:1415-1429 e19.
7. Webster AC, Nagler EV, Morton RL and Masson P. Chronic Kidney Disease. *Lancet*. 2017;389:1238-1252.
8. Marchini J, Howie B, Myers S, McVean G and Donnelly P. A new multipoint method for genome-wide association studies by imputation of genotypes. *Nat Genet*. 2007;39:906-13.
9. Lowe GD, Danesh J, Lewington S, Walker M, Lennon L, Thomson A, Rumley A and Whincup PH. Tissue plasminogen activator antigen and coronary heart disease. Prospective study and meta-analysis. *Eur Heart J*. 2004;25:252-9.
10. Folkersen L, Fauman E, Sabater-Lleal M, Strawbridge RJ, Franberg M, Sennblad B, Baldassarre D, Veglia F, Humphries SE, Rauramaa R, et al. Mapping of 79 loci for 83 plasma protein biomarkers in cardiovascular disease. *PLoS Genet*. 2017;13:e1006706.
11. Gaudet D, Digenio A, Alexander V, Arca M, Jones A, Stroes E, Bergeron J, Civeira F, Hemphill L, Blom D, et al. The approach study: a randomized, double-blind, placebo-controlled, phase 3 study of volanesorsen administered subcutaneously to patients with familial chylomicronemia syndrome (FCS). *Atherosclerosis*. 2017;263:e10.
12. Evangelou E, Warren HR, Mosen-Ansorena D, Mifsud B, Pazoki R, Gao H, Ntritsos G, Dimou N, Cabrera CP, Karaman I, et al. Genetic analysis of over 1 million people identifies 535 new loci associated with blood pressure traits. *Nat Genet*. 2018;50:1412-1425.
13. Burgess S and Thompson SG. Multivariable Mendelian randomization: the use of pleiotropic genetic variants to estimate causal effects. *Am J Epidemiol*. 2015;181:251-60.
14. Burgess S, Butterworth A and Thompson SG. Mendelian randomization analysis with multiple genetic variants using summarized data. *Genet Epidemiol*. 2013;37:658-65.
15. Bowden J, Davey Smith G and Burgess S. Mendelian randomization with invalid instruments: effect estimation and bias detection through Egger regression. *Int J Epidemiol*. 2015;44:512-25.
16. Bowden J, Davey Smith G, Haycock PC and Burgess S. Consistent Estimation in Mendelian Randomization with Some Invalid Instruments Using a Weighted Median Estimator. *Genet Epidemiol*. 2016;40:304-14.
17. Staley JR, Blackshaw J, Kamat MA, Ellis S, Surendran P, Sun BB, Paul DS, Freitag D, Burgess S, Danesh J, et al. PhenoScanner: a database of human genotype-phenotype associations. *Bioinformatics*. 2016;32:3207-3209.
18. Lindstrom S, Wang L, Smith EN, Gordon W, van Hylckama Vlieg A, de Andrade M, Brody JA, Pattee JW, Haessler J, Brumpton BM, et al. Genomic and Transcriptomic Association Studies Identify 16 Novel Susceptibility Loci for Venous Thromboembolism. *Blood*. 2019.
