# Supplementary material for: Genetic Determinants of Lipids and Cardiovascular Disease Outcomes: A Wide-Angled Mendelian Randomization Investigation
Source: Circ Genom Precis Med. 2019 Dec 17;12(12):e002711. doi: 10.1161/CIRCGEN.119.002711 (PMC6922071; doi:10.1161/CIRCGEN.119.002711)
Supplement: Supplementary file 2 [file hcg-12-e002711-s002.pdf]

Genetic Determinants of Lipids and Cardiovascular Disease Outcomes: A Wide-angled Mendelian Randomization Investigation

Running title: *Allara et al.; Genetics of lipids and cardiovascular diseases*

Elias Allara, MD[1,2]; Gabriele Morani, PhD[3]; Paul Carter, MB, BS[1]; Apostolos Gkatzionis, PhD[4]; Verena Zuber, PhD[4,5]; Christopher N. Foley, PhD[4]; Jessica MB Rees, MPhil[1,6]; Amy M. Mason, PhD[1]; Steven Bell, PhD[1,2]; Dipender Gill, MD[5]; Sara Lindstroem, PhD[7]; Adam S. Butterworth, PhD[1,2], Emanuele Di Angelantonio, FRCP[1,2]; James Peters, MB, ChB, PhD[1]; Stephen Burgess, PhD[1,4], and the INVENT consortium

[1]MRC/BHF Cardiovascular Epidemiology Unit, Dept of Public Health & Primary Care, [2]National Institute for Health Research Blood & Transplant Research Unit in Donor Health & Genomics, [4]MRC Biostatistics Unit, Univ of Cambridge, Cambridge, United Kingdom; [3]Dipartimento di Scienze del Sistema Nervoso e del Comportamento, Università degli studi di Pavia, Pavia, Italy; [5]Dept of Epidemiology & Biostatistics, Imperial College London, London, [6]Edinburgh Clinical Trials Unit, Usher Inst of Population Health Sciences & Informatics, Univ of Edinburgh, Edinburgh, United Kingdom; [7]Dept of Epidemiology, Univ of Washington, Seattle, WA

and Precision Medicine

Correspondence:

Stephen Burgess, PhD

MRC Biostatistics Unit

Cambridge Institute of Public Health

Robinson Way

Cambridge, CB2 0SR

United Kingdom

Tel: +44 1223 768259

Email: sb452@medschl.cam.ac.uk

Journal Subject Terms: Lipids and Cholesterol, Cardiovascular Disease, Etiology, Epidemiology, Genetics

Abstract:

Background - Evidence from randomized trials has shown that therapies that lower low-density lipoprotein (LDL)-cholesterol and triglycerides reduce coronary artery disease (CAD) risk. However, there is still uncertainty regarding their effects on other cardiovascular outcomes. We therefore performed a systematic investigation of causal relationships between circulating lipids and cardiovascular outcomes using a Mendelian randomization approach.

Methods - In the primary analysis, we performed two-sample multivariable Mendelian randomization using data from participants of European ancestry. We also conducted univariable analyses using inverse-variance weighted and robust methods, and gene-specific analyses using variants that can be considered as proxies for specific lipid-lowering medications. We obtained associations with lipid fractions from the Global Lipids Genetics Consortium, a meta-analysis of 188,577 participants, and genetic associations with cardiovascular outcomes from 367,703 participants in UK Biobank.

Results - For LDL-cholesterol, in addition to the expected positive associations with CAD risk (odds ratio per 1 standard deviation increase [OR], 1.45; 95% confidence interval [95%CI] 1.35-1.57) and other atheromatous outcomes (ischemic cerebrovascular disease and peripheral vascular disease), we found independent associations of genetically-predicted LDL-cholesterol with abdominal aortic aneurysm (OR 1.75; 95%CI 1.40-2.17) and aortic valve stenosis (OR 1.46; 95%CI 1.25-1.70). Genetically-predicted triglyceride levels were positively associated with CAD (OR 1.25; 95%CI 1.12-1.40), aortic valve stenosis (OR 1.29; 95%CI 1.04-1.61), and hypertension (OR 1.17; 95%CI 1.07-1.27), but inversely associated with venous thromboembolism (OR 0.79; 95%CI 0.67-0.93) and haemorrhagic stroke (OR 0.78; 95%CI 0.62-0.98). We also found positive associations of genetically-predicted LDL-cholesterol and triglycerides with heart failure that appeared to be mediated by CAD.

Conclusions - Lowering LDL-cholesterol is likely to prevent abdominal aortic aneurysm and aortic stenosis, in addition to CAD and other atheromatous cardiovascular outcomes. Lowering triglycerides is likely to prevent CAD and aortic valve stenosis, but may increase thromboembolic risk.

Circulation: Genomic and Precision Medicine

Key words: lipids; cardiovascular outcomes; etiology; epidemiology; Mendelian randomization

Non-standard Abbreviations and Acronyms

APOC3, apolipoprotein C3

BMI, body mass index

CAD, coronary artery disease

HDL-cholesterol, high-density lipoprotein cholesterol

HMGCR, 3-hydroxy-3-methylglutaryl-CoA reductase

INVENT, International Network against Thrombosis

LDL-cholesterol, low-density lipoprotein cholesterol

LDLR, low-density lipoprotein receptor

LPL, lipoprotein lipase

MR, Mendelian randomization

PCSK9, proprotein convertase subtilisin/kexin type 9

T2D, type-2 diabetes

American Heart Association
Circulation: Genomic and Precision Medicine

Introduction

Evidence from randomized trials has shown that therapies that lower low-density lipoprotein (LDL)-cholesterol, such as statins, are beneficial for preventing or treating several atheromatous diseases such as coronary artery disease (CAD),[1] ischaemic stroke,[2] and peripheral vascular disease,[3, 4] as well as both postoperative atrial fibrillation[5] and heart failure in the presence of underlying CAD.[6, 7] However, for other cardiovascular outcomes, such as thromboembolic disease,[8] haemorrhagic stroke[9, 10] and aortic aneurysms,[11, 12] the effects of LDL-cholesterol lowering are less clear. Recently, the REDUCE-IT trial has shown that therapies that predominantly lower triglycerides can reduce major cardiovascular events.[13] However, the effects of triglyceride lowering on non-atheromatous cardiovascular outcomes are largely unknown.

American Heart Association.
Circulation: Genomic and Precision Medicine

Although a randomized trial is the gold standard of evidence and is required to conclusively establish the effectiveness of a treatment, naturally-occurring genetic variants can be used to help predict the outcome of a randomized trial in an approach known as Mendelian randomization.[14] For example, this approach has been used successfully to validate the effects of statins and predict the effects of proprotein convertase subtilisin/kexin type 9 (PCSK9) inhibitors on CAD risk seen in randomized trials.[15, 16] Mendelian randomization investigations have also shown positive associations of genetically-predicted LDL-cholesterol with abdominal aortic aneurysm,[17] ischaemic stroke,[18] and aortic stenosis,[19] and positive associations of triglycerides with CAD.[20] While Mendelian randomization makes strong assumptions that the genetic variants used in the analysis only influence the outcome via the stated risk factors,[21] when both approaches can be undertaken Mendelian randomization has generally given results that agree with randomized trials.[22]

However, Mendelian randomization approaches have not yet been used to investigate the relationship between circulating lipids and some major cardiovascular conditions such as venous thromboembolism and heart failure. Additionally, approaches to estimate the independent effects of LDL-cholesterol, triglycerides, and high-density lipoprotein (HDL)-cholesterol on cardiovascular disease while accounting for genetic pleiotropy have only been performed for a limited set of outcomes.[17, 19, 20]

Our objective in this paper is to assess which cardiovascular outcomes could be treated by lipid-lowering therapies. We performed a systematic Mendelian randomization analysis of atheromatous and non-atheromatous cardiovascular disease outcomes. We considered disease outcomes in a single dataset (UK Biobank) to ensure a consistent approach to the analysis across different outcomes. We carried out polygenic analyses for each of LDL-cholesterol, HDL-cholesterol, and triglycerides, based on all common genetic variants associated with at least one of these risk factors, as well as gene-specific analyses based on variants in or near gene regions that mimic specific pharmaceutical interventions.

American Heart Association
Circulation: Genomic and Precision Medicine

Methods

Data availability

Summary statistics for the genetic associations with lipid fractions were taken from the Global Lipids Genetics Consortium, and are available at http://csg.sph.umich.edu/willer/public/lipids2013.[23, 24] Summary statistics for the genetic associations with outcomes were estimated in UK Biobank, and for replication with venous thromboembolism in the International Network against Thrombosis (INVENT) Consortium (restricting to European descent participants and excluding participants from UK Biobank).

Because of the sensitive nature of some of the data collected for this study, requests to access the datasets from qualified researchers trained in human subject confidentiality protocols may be sent to UK Biobank at https://www.ukbiobank.ac.uk/register-apply,[25] and to the INVENT Consortium by contacting the senior authors and Co-Conveners of the Consortium, Drs. David-Alexandre Tregouet and Nicholas L. Smith.[26]

UK Biobank and all studies in these consortia were approved by their respective institutional review committee and their participants gave informed consent.

The methods are available as supplemental data.

Results

Participant characteristics

American Heart Association
Circulation: Genomic and Precision Medicine

Baseline characteristics of the participants in the UK Biobank are provided in Table 1. Around 46% of participants were men, and the mean age was 57 years. Around 10% were smokers, 93% were alcohol drinkers, and 4% had a history of diabetes at baseline.

Polygenic analyses for all lipid-related variants

Multivariable Mendelian randomization estimates are displayed graphically in Figure 1 and summarized in Supplementary Table S1. Estimates of heterogeneity between the causal estimates from different variants are provided in Supplementary Tables S2 and S3, and scatter plots of genetic associations for selected risk factor/outcome pairs are provided in Supplementary Figure S1. Significant heterogeneity was observed for several outcomes, although this is unsurprising given the number of genetic variants included in the analyses. Associations with the positive control outcome (CAD) were as expected for LDL-cholesterol and triglycerides. There were no associations with the negative control outcome (chronic kidney disease).

We found a strong association of genetically-predicted LDL-cholesterol with CAD risk (odds ratio per 1 standard deviation increase [OR], 1.45; 95% confidence interval [CI] 1.35-1.57), which was in the reference range of a previous study.[20] The strongest associations by magnitude were for abdominal aortic aneurysm (OR 1.75; 95%CI 1.40-2.17), aortic valve stenosis (OR 1.46; 95% CI 1.25-1.70), aortic aneurysm (OR 1.43; 95%CI 1.21-1.68), and heart failure (OR 1.17; 95%CI 1.06-1.28), which all met our threshold for statistical significance ($p<0.003$). We also observed a significant positive association for the combined outcome of ischaemic stroke and transient ischaemic attack (OR 1.14; 95% CI 1.04-1.24). We saw positive associations at a nominal significance level ($0.003 \leq p < 0.05$) for LDL-cholesterol with risk of transient ischaemic attack and peripheral vascular disease. The association with ischaemic stroke was in the positive direction but non-significant (OR 1.10; 95% CI 0.98-1.23).

Genetically-predicted triglyceride levels were strongly associated with increased risk of CAD, consistent with previous results,[20] as well as with increased risk of hypertension (OR 1.17; 95%CI 1.07-1.27). Positive nominal associations were noted also for aortic valve stenosis and heart failure. A nominally-significant inverse association was observed with haemorrhagic stroke, in particular with intracerebral haemorrhage (OR 0.65; 95%CI 0.49-0.86). Inverse nominal associations were observed for triglycerides with all of the thromboembolic diseases we analysed: deep vein thrombosis (OR 0.78; 95%CI 0.65-0.93), pulmonary embolism (OR 0.78; 95%CI 0.64-0.96), and any venous thromboembolism (OR 0.79; 95%CI 0.67-0.93). A similar association with venous thromboembolism was estimated in a separate sample of 129,002 individuals of European ancestry from the INVENT consortium (OR 0.84; 95%CI 0.70-1.01, $p=0.057$) (Table 2). Genetically-predicted HDL-cholesterol was nominally associated with lower risk of coronary heart disease (OR 0.91; 95%CI 0.83-1.00) and hypertension, and more strongly

American Heart Association.
Circulation: Genomic and Precision Medicine

inversely associated with abdominal aortic aneurysm (OR 0.65; 95%CI 0.51-0.85) and intracerebral haemorrhage (OR 0.65; 95%CI 0.51-0.82). In exploratory analyses to validate these findings, genetically-predicted triglyceride levels were positively associated with tissue-type plasminogen activator, but not with platelet count (Supplementary Table S4). Positive associations were observed between genetically-predicted triglycerides and both systolic and diastolic blood pressure, whereas inverse associations with HDL-cholesterol were not replicated (Supplementary Table S5).

Univariable Mendelian randomization estimates for each lipid risk factor in turn are displayed in Supplementary Figures S2-S4 and summarized in Supplementary Tables S6-S8. Similar results were generally obtained from each of the univariable methods as for the multivariable methods, although confidence intervals were slightly wider in some cases, especially for the MR-Egger method. The association between HDL-cholesterol and CAD became null in MR-Egger regression (OR 0.99; 95%CI 0.83-1.19). Similarly, the associations of triglycerides with haemorrhagic stroke and its subtypes attenuated to the null in almost all univariable analyses (Supplementary Table S8). Another notable difference was a positive association of genetically-predicted triglycerides with increased aortic aneurysm risk, particularly for abdominal aortic aneurysm. While the association with aortic aneurysm was not strongly apparent in the multivariable analysis, it was evident for each of the univariable analysis methods.

American Heart Association
Circulation: Genomic and Precision Medicine

We also assessed whether the associations of genetically-predicted lipids with outcomes that are comorbid with CAD (e.g. heart failure) may be mediated via CAD, and similarly for outcomes that are comorbid with type 2 diabetes (T2D), e.g. hypertension. On adjustment for CAD risk (Table 3), the associations of genetically-predicted LDL-cholesterol and triglycerides

with heart failure attenuated sharply, suggesting that their effects on heart failure might be mediated via CAD. As a negative control, we also performed the same analysis for abdominal aortic aneurysm. The association with genetically-predicted LDL-cholesterol attenuated slightly, but was still clearly positive. We also performed multivariable Mendelian randomization analyses excluding participants with CAD for heart failure (2383 remaining cases). This analysis gave null estimates for all lipid fractions (Supplementary Table S9). This suggests that LDL-cholesterol is unlikely to be a causal risk factor for heart failure where there is no comorbidity with CAD. On adjustment for body mass index (BMI) and T2D (Supplementary Table S10), associations between lipids and hypertension, abdominal aortic aneurysm, and haemorrhagic stroke did not change substantially compared to unadjusted analyses, suggesting that neither BMI nor T2D is likely to mediate the effects of lipids on these outcomes. While adjusting for heart failure, heart failure was strongly associated with venous thromboembolism, but associations between lipids and venous thromboembolism did not change markedly (Supplementary Table S10). This confirms that heart failure is an independent risk factor for venous thromboembolism but suggests it is unlikely to mediate the association between triglycerides and venous thromboembolism.

American Heart Association.
Circulation: Genomic and Precision Medicine

Gene-specific analyses for drug proxy variants

Mendelian randomization estimates for specific gene regions are displayed graphically in Figure 2 and summarized in Supplementary Tables S11-S12. All the gene regions analysed showed clear associations with CAD risk, confirming their involvement in cardiovascular disease aetiology and the relevance of existing and proposed lipid-lowering therapies for CAD risk reduction.

Results for the low-density lipoprotein receptor (*LDLR*) gene regions, and to a lesser extent for 3-hydroxy-3-methylglutaryl-CoA reductase (*HMGCR*) and *PCSK9*, were similar to those for LDL-cholesterol in the polygenic analyses, although with wider confidence intervals. Significant positive associations were obtained for *LDLR* with aortic and abdominal aortic aneurysm, venous thromboembolism, aortic valve stenosis, and heart failure, and an inverse association was observed for subarachnoid haemorrhage. Variants in the *HMGCR* gene region were inversely associated with intracerebral haemorrhage (*p*=0.022). Results for apolipoprotein C3 (*APOC3*) and lipoprotein lipase (*LPL*) followed a pattern more similar to the analysis for triglycerides, showing inverse associations with thromboembolic diseases (*p*=0.007 for *APOC3*, *p*=0.089 for *LPL* for any venous thromboembolism). In both regions, associations with aortic aneurysm, aortic valve stenosis and hypertension were all positive. Additionally, variants in the *LPL* region were also positively associated with all ischaemic cerebrovascular diseases.

American Heart Association
Circulation: Genomic and Precision Medicine

Discussion

In this study, we assessed the causal role of three major lipid fractions for a range of cardiovascular diseases in a large population-based cohort using the principle of Mendelian randomization while accounting for genetic pleiotropy between the lipid measures. Our most notable findings were the associations between genetically-predicted triglycerides and decreased risk of thromboembolic diseases both in polygenic analyses and in gene-specific analyses for the *APOC3* gene region, suggesting that reducing triglycerides may increase risk for venous thromboembolism. Additionally, as summarized in Supplementary Table S13, we found: 1) evidence supporting the current understanding of the aetiology of CAD, suggesting independent causal roles for LDL-cholesterol and triglycerides, both in the polygenic analyses and for all the

drug-related gene-specific analyses[20, 27] 2) positive associations of genetically-predicted LDL-cholesterol with abdominal aortic aneurysm and aortic valve stenosis, as well as atheromatous cardiovascular outcomes that are already addressed in clinical guidelines (e.g. peripheral vascular disease and the combined outcome of ischaemic stroke and transient ischaemic attack); 3) positive associations of genetically-predicted LDL-cholesterol and triglycerides with heart failure that appear to be mediated by CAD; 4) associations between genetically-predicted triglycerides and increased risk of aortic stenosis and hypertension; and 5) inverse associations between genetically-predicted HDL-cholesterol and abdominal aortic aneurysm and haemorrhagic stroke (in particular, intracerebral haemorrhage) that appear to be independent of BMI/T2D.

American Heart Association.
Circulation: Genomic and Precision Medicine

The importance of these findings is threefold. Firstly, they help us to better understand the aetiology and pathophysiology of common cardiovascular outcomes, and so to identify additional potential indications for lipid-lowering therapies. Secondly, association estimates provided in this paper can inform calculations on the risk-benefit and cost-benefit of these therapies. Thirdly, these findings can aid identification of which patients would most benefit from (or should avoid) lipid-lowering therapies.

Genetic predisposition to lower triglycerides was associated with an increased risk of thromboembolic diseases. In contrast, previous observational evidence did not suggest an association between triglycerides and venous thrombosis,[28, 29] although other lipid measures (in particular apolipoprotein B and lipoprotein(a)) were inversely associated with venous thrombosis mortality in a meta-analysis of over 700,000 participants from the Emerging Risk Factors Collaboration.[29] Our result was consistent across 3 of the 4 Mendelian randomization methods and was replicated using data from the INVENT collaboration, an independent data source for

genetic associations with the outcome. Associations with thromboembolic outcomes using the MR-Egger method were slightly attenuated and compatible with the null, raising the possibility of unmeasured genetic pleiotropy, although this method generally has lower power compared to the conventional inverse-variance weighted method.[30] Variants in the *APOC3* gene region were associated with thromboembolic events, suggesting that lowering triglyceride levels via intervening on this pathway may increase thromboembolism risk. Additionally, the positive genetic association between triglycerides and tissue-type plasminogen activator reported here confirms the findings of a previous observational study among 1227 men free of coronary heart disease.[31] However, genetically-predicted triglycerides were not associated with platelet count. This is in contrast to the APPROACH trial, in which thrombocytopenia led to excess early terminations among patients with familial chylomicronaemia[32] but in line with (i) two other trials of APOC3 inhibitors[33, 34] and (ii) with the fluctuations in platelet counts (both thrombocytopenia and thrombocytosis) noted in an observational study of 86 patients with familial chylomicronaemia.[35] Further investigation is needed to confirm the unexpected genetic association of triglycerides with venous thromboembolism.

American Heart Association
Circulation: Genomic and Precision Medicine

Both the positive association between genetic predictors of LDL-cholesterol and abdominal aortic aneurysm, and the inverse association for HDL-cholesterol, replicate a previous Mendelian randomization study which did not include UK Biobank participants.[17] This evidence is complemented by a randomized trial that has shown benefit from screening and treating abdominal aortic aneurysm using various interventions including statins.[11] No significant association was seen with thoracic aortic aneurysm, in line with the different pathophysiology of the two disease subtypes.[36] Overall, these findings provide further support to the hypothesis that increased LDL-cholesterol has deleterious effects on abdominal aortic aneurysm, and suggest

that LDL-cholesterol lowering therapies may be beneficial in preventing abdominal aortic aneurysm.

Similarly, the positive association between genetically-predicted LDL-cholesterol and aortic valve stenosis replicates a previous Mendelian randomization study.[19] We also demonstrated a positive association for triglycerides, in line with the previous study (although their result did not achieve conventional levels of statistical significance). Our association was robust to all sensitivity analyses and evidenced in gene-specific analyses for both the *APOC3* and *LPL* loci. These findings are consistent with previous observational and pathological studies suggesting a role of atherosclerotic processes in early valve lesions[37, 38] whereas clinical trials showed no benefit from LDL-cholesterol lowering via statins on aortic stenosis progression.[39] Taken together, this suggests that increased LDL-cholesterol and triglycerides may facilitate initiation of early lesions, but that lipid-lowering therapies may be ineffective in preventing the progression of aortic stenosis.

American Heart Association.
Circulation: Genomic and Precision Medicine

CAD is a well-known risk factor for heart failure,[40] as myocardial ischaemic damage reduces myocardial contractility and ventricular function. Associations of heart failure with genetically-predicted LDL-cholesterol and triglycerides disappeared after adjusting for CAD. The association was also absent when omitting participants with a CAD diagnosis from analyses. In contrast, associations with abdominal aortic aneurysm attenuated only slightly after adjustment for CAD. Overall, these results suggest that lipid-lowering therapies are only likely to influence risk of heart failure via their effects on CAD.

Our analyses also identified inverse associations of genetically-predicted HDL-C and triglyceride levels with risk of intracerebral haemorrhage (albeit the latter association was not robust to most sensitivity analyses), consistent with a recent observational analysis for

triglycerides.[41] Furthermore, we identified a detrimental effect of HMGCR inhibition on intracerebral haemorrhage risk, in keeping with a large randomized trial of atorvastatin in patients with recent transient ischaemic attack or stroke, which found statin therapy to lower risk of major cardiovascular events but increase the risk of haemorrhagic stroke.[42] However, this finding has not been replicated in large meta-analyses.[43] The mechanism relating lipid traits with risk of intracerebral haemorrhage requires further exploration.

Finally, the inverse association between HDL-cholesterol and CAD was only nominally significant in the main multivariable analyses but became null with MR-Egger regression, suggesting that HDL-cholesterol is unlikely to be a causal risk factor for CAD.

Our investigation has both strengths and limitations. The large sample size of over 360,000 participants and the broad set of outcomes analysed render this one of the most comprehensive Mendelian randomization analyses on cardiovascular disease conducted to date. Availability of multiple cardiovascular conditions within the same study enabled cross-comparisons between diseases, and enabled us to perform adjusted analyses in the same participants and to assess mediation of causal effects. This investigation has a number of limitations. Lack of publicly-available summary data from external datasets prevented us from performing replication analyses for some associations, e.g. those for heart failure. This design still allows sensitivity analyses such as MR-Egger and the weighted median estimator which enabled us to investigate and account for the possible presence of genetic pleiotropy, and gene-specific analyses that are less likely to be influenced by pleiotropy as they only include variants from a single gene region where the function is well-known. Significant heterogeneity was observed in the polygenic analysis for several outcomes, suggesting that some variants may have pleiotropic effects. Additionally, this investigation was conducted in UK-based middle- to late-

American Heart Association.
Circulation: Genomic and Precision Medicine

aged participants of European ancestries. While this is recommended for Mendelian randomization to ensure that genetic associations are not influenced by population stratification, it means that results may not be generalizable to other ethnicities or nationalities.

In conclusion, multivariable Mendelian randomization analyses accounting for lipid-related genetic pleiotropy support the hypothesis that circulating lipids have causal effects on a wide range of cardiovascular diseases. Interventions that lower LDL-cholesterol are likely to be beneficial in preventing aortic aneurysm and aortic stenosis in addition to several other atheromatous diseases. Triglyceride lowering treatments are likely to be beneficial in preventing coronary artery disease and aortic valve stenosis, but caution is needed due to the possibility of increased risk of venous thromboembolism.

American Heart Association
Circulation: Genomic and Precision Medicine

Acknowledgments: The research has been conducted using the UK Biobank Resource under Application Number 26865.

Sources of Funding: The study's coordinating centre has been underpinned by grants G0800270, MR/L003120/1 and MC_UU_12013/3 from the UK Medical Research Council, grants SP/09/002, RG/08/014, and RG13/13/30194 from the British Heart Foundation, grants from the National Institute for Health Research (NIHR) through the Cambridge Biomedical Research Centre, and grant HEALTH-F2-2012-279233 from the European Commission Framework 7 through the EPIC-CVD award. The NIHR Blood and Transplant Research Unit (BTRU) in Donor Health and Genomics is supported by grant NIHR BTRU-2014-10024. Dr Burgess is supported by a Sir Henry Dale Fellowship jointly funded by the Wellcome Trust and the Royal Society (grant number 204623/Z/16/Z). Dr Allara is supported by a NIHR BTRU PhD Studentship. Dr Gill is funded by the Wellcome 4i Clinical PhD Programme at Imperial College London. Dr Peters was funded by a UKRI Innovation Fellowship (MR/S004068/1). This work has received support from the EU/EFPIA Innovative Medicines Initiative (https://www.imi.europa.eu/) Joint Undertaking BigData@Heart grant n° 116074. Aspects of the

analysis were supported by the Cambridge Substantive Site of Health Data Research UK and the National Institute for Health Research (Cambridge Biomedical Research Centre at the Cambridge University Hospitals NHS Foundation Trust) [*]. The funding sources had no role in the design and conduct of the study; collection, management, analysis, and interpretation of the data; preparation, review, or approval of the manuscript; and decision to submit the manuscript for publication.
*The views expressed are those of the authors and not necessarily those of the NHS, the NIHR or the Department of Health and Social Care.

Disclosures: None.

References:

1. Cholesterol Treatment Trialists C, Mihaylova B, Emberson J, Blackwell L, Keech A, Simes J, Barnes EH, Voysey M, Gray A, Collins R, et al. The effects of lowering LDL cholesterol with statin therapy in people at low risk of vascular disease: meta-analysis of individual data from 27 randomised trials. *Lancet*. 2012;380:581-90.

2. Kernan WN, Ovbiagele B, Black HR, Bravata DM, Chimowitz MI, Ezekowitz MD, Fang MC, Fisher M, Furie KL, Heck DV, et al. Guidelines for the prevention of stroke in patients with stroke and transient ischemic attack: a guideline for healthcare professionals from the American Heart Association/American Stroke Association. *Stroke*. 2014;45:2160-236.

3. Gerhard-Herman MD, Gornik HL, Barrett C, Barshes NR, Corriere MA, Drachman DE, Fleisher LA, Fowkes FG, Hamburg NM, Kinlay S, et al. 2016 AHA/ACC Guideline on the Management of Patients With Lower Extremity Peripheral Artery Disease: A Report of the American College of Cardiology/American Heart Association Task Force on Clinical Practice Guidelines. *J Am Coll Cardiol*. 2017;69:e71-e126.

4. Aboyans V, Ricco JB, Bartelink MEL, Bjorck M, Brodmann M, Cohnert T, Collet JP, Czerny M, De Carlo M, Debus S, et al. 2017 ESC Guidelines on the Diagnosis and Treatment of Peripheral Arterial Diseases, in collaboration with the European Society for Vascular Surgery (ESVS): Document covering atherosclerotic disease of extracranial carotid and vertebral, mesenteric, renal, upper and lower extremity arteriesEndorsed by: the European Stroke Organization (ESO)The Task Force for the Diagnosis and Treatment of Peripheral Arterial Diseases of the European Society of Cardiology (ESC) and of the European Society for Vascular Surgery (ESVS). *Eur Heart J*. 2018;39:763-816.

5. January CT, Wann LS, Alpert JS, Calkins H, Cigarroa JE, Cleveland JC, Jr., Conti JB, Ellinor PT, Ezekowitz MD, Field ME, et al. 2014 AHA/ACC/HRS guideline for the management of patients with atrial fibrillation: a report of the American College of Cardiology/American Heart

Circulation: Genomic and Precision Medicine

Association Task Force on Practice Guidelines and the Heart Rhythm Society. *J Am Coll Cardiol*. 2014;64:e1-76.

6. Yancy CW, Jessup M, Bozkurt B, Butler J, Casey DE, Jr., Drazner MH, Fonarow GC, Geraci SA, Horwich T, Januzzi JL, et al. 2013 ACCF/AHA guideline for the management of heart failure: a report of the American College of Cardiology Foundation/American Heart Association Task Force on Practice Guidelines. *J Am Coll Cardiol*. 2013;62:e147-239.

7. Ponikowski P, Voors AA, Anker SD, Bueno H, Cleland JG, Coats AJ, Falk V, Gonzalez-Juanatey JR, Harjola VP, Jankowska EA, et al. 2016 ESC Guidelines for the diagnosis and treatment of acute and chronic heart failure: The Task Force for the diagnosis and treatment of acute and chronic heart failure of the European Society of Cardiology (ESC). Developed with the special contribution of the Heart Failure Association (HFA) of the ESC. *Eur J Heart Fail*. 2016;18:891-975.

8. Kunutsor SK, Seidu S, Khunti K. Statins and primary prevention of venous thromboembolism: a systematic review and meta-analysis. *Lancet Haematol*. 2017;4:e83-e93.

9. Van Matre ET, Sherman DS, Kiser TH. Management of intracerebral hemorrhage--use of statins. *Vasc Health Risk Manag*. 2016;12:153-61.

10. Ma Y, Li Z, Chen L, Li X. Blood lipid levels, statin therapy and the risk of intracerebral hemorrhage. *Lipids Health Dis*. 2016;15:43.

11. Lindholt JS, Sogaard R. Population screening and intervention for vascular disease in Danish men (VIVA): a randomised controlled trial. *Lancet*. 2017;390:2256-2265.

12. Stein LH, Berger J, Tranquilli M, Elefteraides JA. Effect of statin drugs on thoracic aortic aneurysms. *Am J Cardiol*. 2013;112:1240-5.

13. Bhatt DL, Steg PG, Miller M, Brinton EA, Jacobson TA, Ketchum SB, Doyle RT, Jr., Juliano RA, Jiao L, Granowitz C, et al. Cardiovascular Risk Reduction with Icosapent Ethyl for Hypertriglyceridemia. *N Engl J Med*. 2018.

14. Smith GD, Ebrahim S. 'Mendelian randomization': can genetic epidemiology contribute to understanding environmental determinants of disease? *Int J Epidemiol*. 2003;32:1-22.

15. Ference BA, Robinson JG, Brook RD, Catapano AL, Chapman MJ, Neff DR, Voros S, Giugliano RP, Davey Smith G, Fazio S, et al. Variation in PCSK9 and HMGCR and Risk of Cardiovascular Disease and Diabetes. *N Engl J Med*. 2016;375:2144-2153.

16. Cohen JC, Boerwinkle E, Mosley TH, Jr., Hobbs HH. Sequence variations in PCSK9, low LDL, and protection against coronary heart disease. *N Engl J Med*. 2006;354:1264-72.

American Heart Association.
Circulation: Genomic and Precision Medicine

17. Harrison SC, Holmes MV, Burgess S, Asselbergs FW, Jones GT, Baas AF, van 't Hof FN, de Bakker PIW, Blankensteijn JD, Powell JT, et al. Genetic Association of Lipids and Lipid Drug Targets With Abdominal Aortic Aneurysm: A Meta-analysis. *JAMA Cardiol*. 2018;3:26-33.

18. Hindy G, Engstrom G, Larsson SC, Traylor M, Markus HS, Melander O, Orho-Melander M and Stroke Genetics N. Role of Blood Lipids in the Development of Ischemic Stroke and its Subtypes: A Mendelian Randomization Study. *Stroke*. 2018;49:820-827.

19. Smith JG, Luk K, Schulz CA, Engert JC, Do R, Hindy G, Rukh G, Dufresne L, Almgren P, Owens DS, et al. Association of low-density lipoprotein cholesterol-related genetic variants with aortic valve calcium and incident aortic stenosis. *JAMA*. 2014;312:1764-71.

20. Holmes MV, Asselbergs FW, Palmer TM, Drenos F, Lanktree MB, Nelson CP, Dale CE, Padmanabhan S, Finan C, Swerdlow DI, et al. Mendelian randomization of blood lipids for coronary heart disease. *Eur Heart J*. 2015;36:539-50.

21. Lawlor DA, Harbord RM, Sterne JA, Timpson N, Davey SG. Mendelian randomization: using genes as instruments for making causal inferences in epidemiology. *Stat Med*. 2008;27:1133-1163.

22. Davey Smith G, Hemani G. Mendelian randomization: genetic anchors for causal inference in epidemiological studies. *Hum Mol Genet*. 2014;23:R89-98.

23. Do R, Willer CJ, Schmidt EM, Sengupta S, Gao C, Peloso GM, Gustafsson S, Kanoni S, Ganna A, Chen J, et al. Common variants associated with plasma triglycerides and risk for coronary artery disease. *Nat Genet*. 2013;45:1345-52.

24. Willer CJ, Schmidt EM, Sengupta S, Peloso GM, Gustafsson S, Kanoni S, Ganna A, Chen J, Buchkovich ML, Mora S, et al. Discovery and refinement of loci associated with lipid levels. *Nat Genet*. 2013;45:1274-1283.

25. Sudlow C, Gallacher J, Allen N, Beral V, Burton P, Danesh J, Downey P, Elliott P, Green J, Landray M, et al. UK biobank: an open access resource for identifying the causes of a wide range of complex diseases of middle and old age. *PLoS Med*. 2015;12:e1001779.

26. Lindstrom S, Wang L, Smith EN, Gordon W, van Hylckama Vlieg A, de Andrade M, Brody JA, Pattee JW, Haessler J, Brumpton BM, et al. Genomic and Transcriptomic Association Studies Identify 16 Novel Susceptibility Loci for Venous Thromboembolism. *Blood*. 2019.

27. Voight BF, Peloso GM, Orho-Melander M, Frikke-Schmidt R, Barbalic M, Jensen MK, Hindy G, Holm H, Ding EL, Johnson T, et al. Plasma HDL cholesterol and risk of myocardial infarction: a mendelian randomisation study. *Lancet*. 2012;380:572-80.

28. Morelli VM, Lijfering WM, Bos MHA, Rosendaal FR, Cannegieter SC. Lipid levels and risk of venous thrombosis: results from the MEGA-study. *Eur J Epidemiol*. 2017;32:669-681.

American Heart Association
Circulation: Genomic and Precision Medicine

29. Gregson J, Kaptoge S, Bolton T, Pennells L, Willeit P, Burgess S, Bell S, Sweeting M, Rimm EB, Kabrhel C, et al. Cardiovascular Risk Factors Associated With Venous Thromboembolism. *JAMA Cardiol*. 2019;4:163-173.

30. Bowden J, Davey Smith G, Burgess S. Mendelian randomization with invalid instruments: effect estimation and bias detection through Egger regression. *Int J Epidemiol*. 2015;44:512-25.

31. Lowe GD, Danesh J, Lewington S, Walker M, Lennon L, Thomson A, Rumley A, Whincup PH. Tissue plasminogen activator antigen and coronary heart disease. Prospective study and meta-analysis. *Eur Heart J*. 2004;25:252-9.

32. Gaudet D, Digenio A, Alexander V, Arca M, Jones A, Stroes E, Bergeron J, Civeira F, Hemphill L, Blom D, et al. The approach study: a randomized, double-blind, placebo-controlled, phase 3 study of volanesorsen administered subcutaneously to patients with familial chylomicronemia syndrome (FCS). *Atherosclerosis*. 2017;263:e10.

33. Gouni-Berthold I, Alexander VJ, Digenio A, DuFour R, Steinhagen-Thiessen E, Martin S, Moriarty P, Hughes S, Gaudet D, Stroes E, et al. Apolipoprotein C-III Inhibition with Volanesorsen in Patients with Hypertriglyceridemia (COMPASS): a Randomized, Double-Blind, Placebo-controlled Trial. *Atherosclerosis Supplements*. 2018;32:25.

34. Alexander VJ, Xia S, Hurh E, Hughes SG, O'Dea L, Geary RS, Witztum JL, Tsimikas S. N-acetyl galactosamine-conjugated antisense drug to APOC3 mRNA, triglycerides and atherogenic lipoprotein levels. *Eur Heart J*. 2019.

35. Gaudet D, Baass A, Tremblay K, Brisson D, Laflamme N, Paquette M, Dufour R, Bergeron J. Natural History (up to 15 years) of Platelet Count in 84 Patients with Familial Hyperchylomicronemia Due to Lipoprotein Lipase Deficiency. *Journal of Clinical Lipidology*. 2017;11:797-798.

36. Isselbacher EM. Thoracic and abdominal aortic aneurysms. *Circulation*. 2005;111:816-28.

37. Paradis JM, Fried J, Nazif T, Kirtane A, Harjai K, Khalique O, Grubb K, George I, Hahn R, Williams M, et al. Aortic stenosis and coronary artery disease: what do we know? What don't we know? A comprehensive review of the literature with proposed treatment algorithms. *Eur Heart J*. 2014;35:2069-82.

38. Pibarot P, Dumesnil JG. Improving assessment of aortic stenosis. *J Am Coll Cardiol*. 2012;60:169-80.

39. Zhao Y, Nicoll R, He YH, Henein MY. The effect of statins on valve function and calcification in aortic stenosis: A meta-analysis. *Atherosclerosis*. 2016;246:318-24.

40. Kemp CD, Conte JV. The pathophysiology of heart failure. *Cardiovasc Pathol*. 2012;21:365-71.

41. Sun L, Clarke R, Bennett D, Guo Y, Walters RG, Hill M, Parish S, Millwood IY, Bian Z, Chen Y, et al. Causal associations of blood lipids with risk of ischemic stroke and intracerebral hemorrhage in Chinese adults. *Nat Med*. 2019;25:569-574.

42. Amarenco P, Bogousslavsky J, Callahan A, 3rd, Goldstein LB, Hennerici M, Rudolph AE, Sillesen H, Simunovic L, Szarek M, Welch KM, et al. High-dose atorvastatin after stroke or transient ischemic attack. *N Engl J Med*. 2006;355:549-59.

43. Ziff OJ, Banerjee G, Ambler G, Werring DJ. Statins and the risk of intracerebral haemorrhage in patients with stroke: systematic review and meta-analysis. *J Neurol Neurosurg Psychiatry*. 2019;90:75-83.

American Heart Association
Circulation: Genomic and Precision Medicine

Table 1: Baseline characteristics of UK Biobank participants included in this study and numbers of outcome events

Characteristic	Mean (SD) or N (%)
Sample size	367,703 (100)
Male	168,799 (45.9)
Age at baseline	57.2 (8.1)
Body mass index	27.3 (4.8)
Systolic blood pressure	137.6 (18.6)
Diastolic blood pressure	82.0 (10.1)
Smoking status (current / ex / never)*	37,866 (10.3) / 185,704 (50.5) / 143,777 (39.1)
Alcohol status (current / ex / never)*	342,797 (93.2) / 12,732 (3.5) / 11,646 (3.2)
History of type 2 diabetes	15,834 (4.3)
Coronary Artery Disease	29,278 (8.0)
Ischaemic Cerebrovascular Event (all)	8084 (2.2)
- Ischaemic Stroke	4602 (1.3)
- Transient Ischaemic Attack	3962 (1.1)
Haemorrhagic Stroke (all)	1981 (0.5)
- Intracerebral Haemorrhage	1064 (0.3)
- Subarachnoid Haemorrhage	1084 (0.3)
Aortic Aneurysm (all)	1849 (0.5)
- Abdominal Aortic Aneurysm	1094 (0.3)
- Thoracic Aortic Aneurysm	347 (0.1)
Venous Thromboembolism (all)	14,097 (3.8)
- Deep Vein Thrombosis	9454 (2.6)
- Pulmonary Embolism	6148 (1.7)
Hypertension	125,846 (34.2)
Peripheral Vascular Disease	3415 (0.9)
Aortic Valve Stenosis	2244 (0.6)
Atrial Fibrillation	16,945 (4.6)
Heart Failure	6712 (1.8)
Chronic Kidney Disease	6321 (1.7)

* Excluding 356 participants with smoking status absent and 528 participants with alcohol consumption status absent

American Heart Association.
Circulation: Genomic and Precision Medicine

Table 2: Replication and meta-analysis of the association between genetically-predicted triglycerides and risk of venous thromboembolism in the INVENT consortium.

Venous thromboembolism	**N events / participants**	**HDL-cholesterol**	**LDL-cholesterol**	**Triglycerides**
UK Biobank	14,097 / 367,703	0.94 (0.82-1.08)	1.03 (0.92-1.16)	0.79 (0.67-0.93)*
INVENT	15,572 / 129,002	1.03 (0.89-1.20)	1.07 (0.94-1.21)	0.84 (0.70-1.01)
UK Biobank + INVENT	29,669 / 496,705	0.98 (0.86-1.12)	1.05 (0.94-1.17)	0.81 (0.69-0.95)*

*: $p < 0.05$ †: $p < 0.003$ ‡: $p < 10^{-8}$
All estimates are odds ratios estimated using multivariable inverse-variance weighted Mendelian randomization accounting for lipid-related genetic pleiotropy and between-variant heterogeneity (random-effects analyses).

American Heart Association. Circulation: Genomic and Precision Medicine

Table 3: Multivariable Mendelian randomization estimates (odds ratio with 95% confidence interval) for heart failure without and with adjustment for coronary artery disease (CAD). Analyses were also performed for abdominal aortic aneurysm as a negative control.

Heart failure	**HDL-cholesterol**	**LDL-cholesterol**	**Triglycerides**	**CAD**
Without adjustment	0.97 (0.87-1.08)	1.17 (1.06-1.28)†	1.19 (1.04-1.37)*	-
With adjustment	1.02 (0.92-1.13)	0.95 (0.85-1.06)	1.06 (0.93-1.20)	1.73 (1.47-2.04)‡

Abdominal aortic aneurysm	**HDL-cholesterol**	**LDL-cholesterol**	**Triglycerides**	**CAD**
Without adjustment	0.65 (0.51-0.85)†	1.75 (1.40-2.17)†	0.94 (0.68-1.28)	-
With adjustment	0.69 (0.53-0.88)†	1.46 (1.12-1.91)†	0.84 (0.61-1.16)	1.60 (1.06-2.42)*

*: $p < 0.05$ †: $p < 0.003$ ‡: $p < 10^{-8}$

The CAD column indicates the association between genetically-predicted coronary artery disease and the outcome of interest (i.e. heart failure or abdominal aortic aneurysm) while accounting for the genetic associations of the lipid fractions in a multivariable analysis. If the regression coefficients for the lipid measurements remain unchanged on adjustment for CAD, then the effects of lipids on the outcome do not operate via CAD. If the regression coefficients attenuate to the null, then the effects of lipids on the outcome are entirely mediated via CAD.

Circulation: Genomic and Precision Medicine
American Heart Association

Figure Legends:

Figure 1: Multivariable Mendelian randomization estimates (odds ratio with 95% confidence interval per 1 standard deviation increase in lipid fraction) from polygenic analyses including all lipid-associated variants

Figure 2: Univariable Mendelian randomization estimates (odds ratio with 95% confidence interval per 1 standard deviation increase in lipid fraction) for variants in specific gene regions. Estimates are scaled to a unit standard deviation increase in LDL-cholesterol for the *HMGCR*, *PCSK9*, and *LDLR* regions, and to a standard deviation increase in triglycerides for the *APOC3* and *LPL* regions.

American Heart Association.
Circulation: Genomic and Precision Medicine

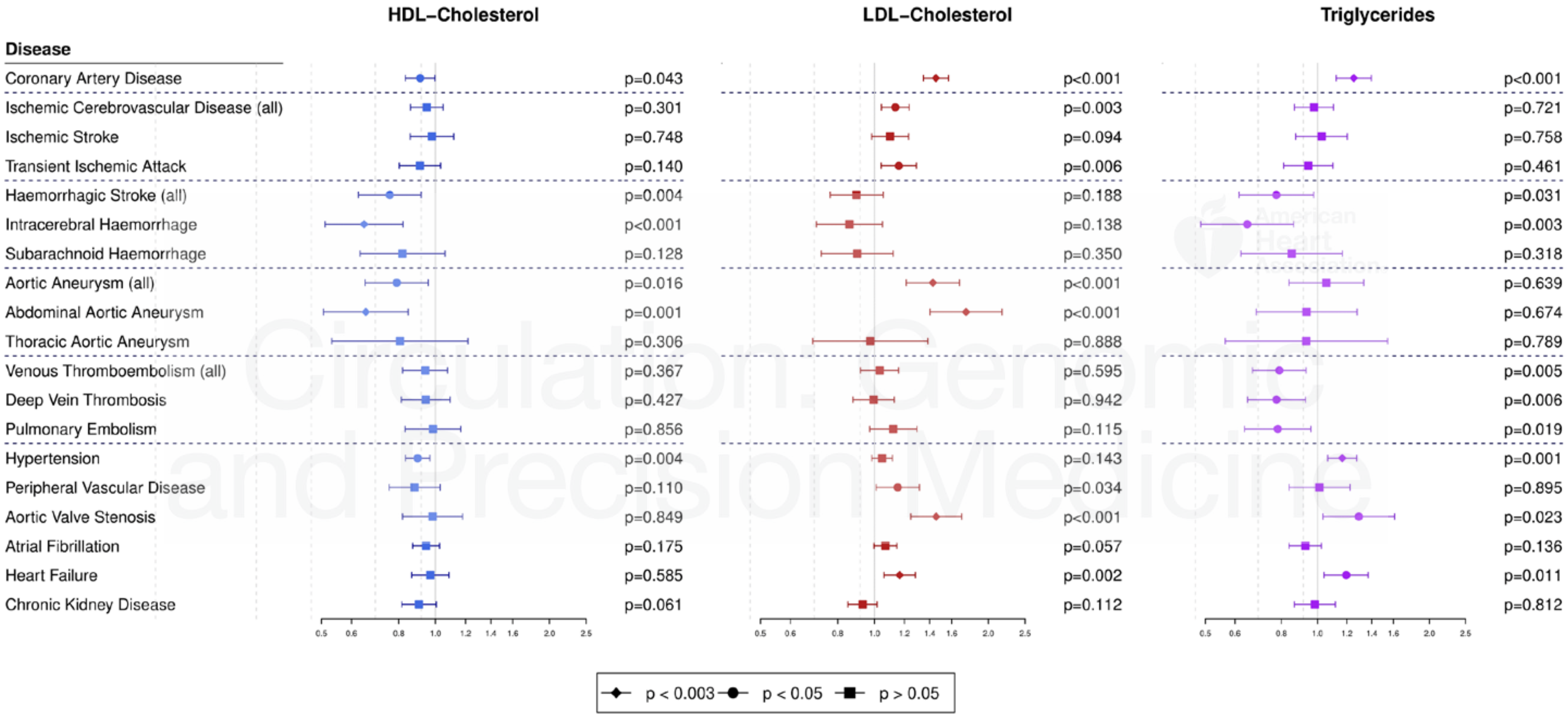
HDL−Cholesterol
LDL−Cholesterol
Triglycerides
Disease
Coronary Artery Disease
Ischemic Cerebrovascular Disease (all)
Ischemic Stroke
Transient Ischemic Attack
Haemorrhagic Stroke (all)
Intracerebral Haemorrhage
Subarachnoid Haemorrhage
Aortic Aneurysm (all)
Abdominal Aortic Aneurysm
Thoracic Aortic Aneurysm
Venous Thromboembolism (all)
Deep Vein Thrombosis
Pulmonary Embolism
Hypertension
Peripheral Vascular Disease
Aortic Valve Stenosis
Atrial Fibrillation
Heart Failure
Chronic Kidney Disease
p=0.043
p=0.301
p=0.748
p=0.140
p=0.004
p<0.001
p=0.128
p=0.016
p=0.001
p=0.306
p=0.367
p=0.427
p=0.856
p=0.004
p=0.110
p=0.849
p=0.175
p=0.585
p=0.061
p<0.001
p=0.003
p=0.094
p=0.006
p=0.188
p=0.138
p=0.350
p<0.001
p<0.001
p=0.888
p=0.595
p=0.942
p=0.115
p=0.143
p=0.034
p<0.001
p=0.057
p=0.002
p=0.112
p<0.001
p=0.721
p=0.758
p=0.461
p=0.031
p=0.003
p=0.318
p=0.639
p=0.674
p=0.789
p=0.005
p=0.006
p=0.019
p=0.001
p=0.895
p=0.023
p=0.136
p=0.011
p=0.812
0.5 0.6 0.8 1.0 1.2 1.4 1.6 2.0 2.5
p < 0.003
p < 0.05
p > 0.05

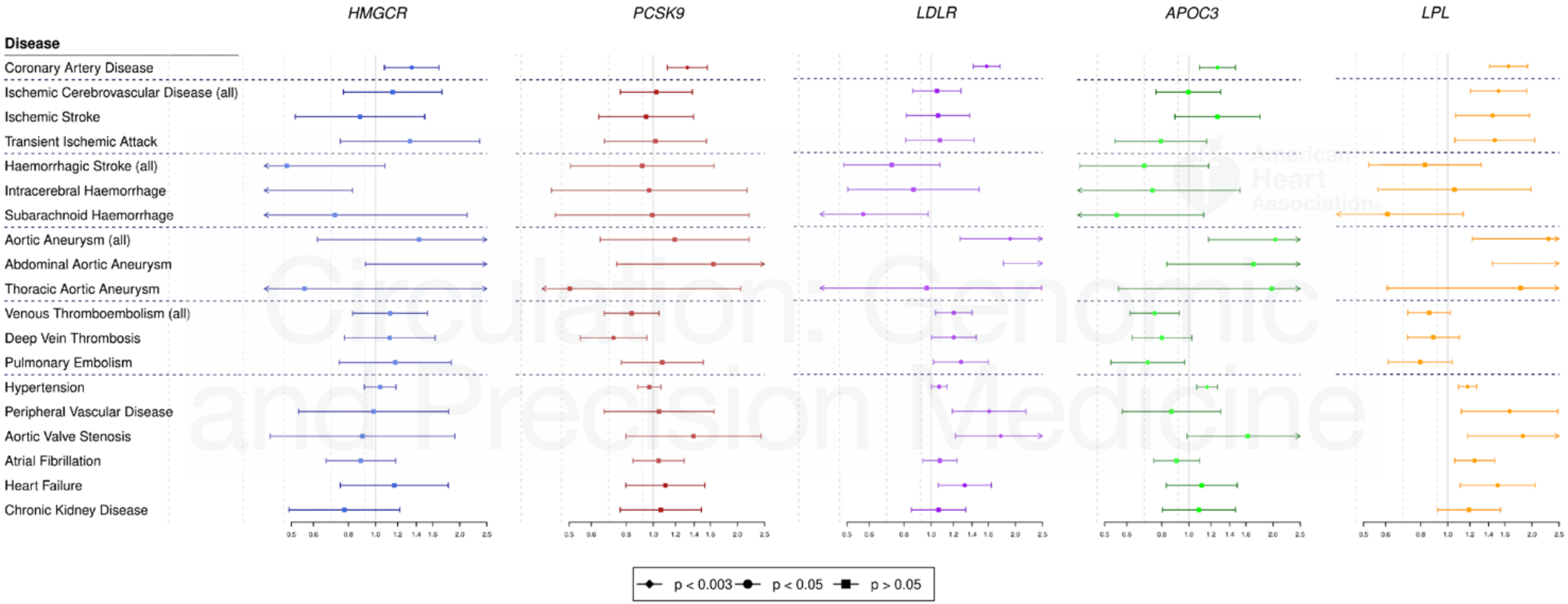

HMGCR
PCSK9
LDLR
APOC3
LPL
Disease
Coronary Artery Disease
Ischemic Cerebrovascular Disease (all)
Ischemic Stroke
Transient Ischemic Attack
Haemorrhagic Stroke (all)
Intracerebral Haemorrhage
Subarachnoid Haemorrhage
Aortic Aneurysm (all)
Abdominal Aortic Aneurysm
Thoracic Aortic Aneurysm
Venous Thromboembolism (all)
Deep Vein Thrombosis
Pulmonary Embolism
Hypertension
Peripheral Vascular Disease
Aortic Valve Stenosis
Atrial Fibrillation
Heart Failure
Chronic Kidney Disease
p < 0.003
p < 0.05
p > 0.05
Circulation: Genomic and Precision Medicine
American Heart Association
